# A novel cyanobacterial geosmin producer, revising *Geo*A distribution and dispersion patterns in Bacteria

**DOI:** 10.1038/s41598-020-64774-y

**Published:** 2020-05-26

**Authors:** Catarina Churro, Ana P. Semedo-Aguiar, Alexandra D. Silva, Jose B. Pereira-Leal, Ricardo B. Leite

**Affiliations:** 1 Laboratório de Fitoplâncton, Departamento do Mar e Recursos Marinhos, Instituto Português do Mar e da Atmosfera, Rua Alfredo Magalhães Ramalho, 6, 1449-006 Lisboa, Portugal; 2 Blue Biotechnology and Ecotoxicology (BBE), CIIMAR - Centro Interdisciplinar de Investigação Marinha e Ambiental, Universidade do Porto, 4450-208 Matosinhos, Portugal; 3 Instituto Gulbenkian de Ciência, Rua da Quinta Grande n°6, 2780-156 Oeiras, Portugal; 4 Programa de Pós-Graduação Ciência para o Desenvolvimento, Rua da Quinta Grande n°6, 2780-156 Oeiras, Portugal; 5 Universidade Jean Piaget de Cabo Verde, Campus da Praia, Caixa Postal 775, Palmarejo Grande, Praia, Cabo Verde; 6 Ophiomics-Precision Medicine, Pólo Tecnológico de Lisboa, Rua Cupertino de Miranda, 9, Lote 8, 1600-513 Lisbon, Portugal

**Keywords:** Genome, Soil microbiology

## Abstract

Cyanobacteria are ubiquitous organisms with a relevant contribution to primary production in all range of habitats. Cyanobacteria are well known for their part in worldwide occurrence of aquatic blooms while producing a myriad of natural compounds, some with toxic potential, but others of high economical impact, as geosmin. We performed an environmental survey of cyanobacterial soil colonies to identify interesting metabolic pathways and adaptation strategies used by these microorganisms and isolated, sequenced and assembled the genome of a cyanobacterium that displayed a distinctive earthy/musty smell, typical of geosmin, confirmed by GC-MS analysis of the culture’s volatile extract. Morphological studies pointed to a new Oscillatoriales soil ecotype confirmed by phylogenetic analysis, which we named *Microcoleus asticus* sp. nov. Our studies of geosmin gene presence in Bacteria, revealed a scattered distribution among Cyanobacteria, Actinobacteria, Delta and Gammaproteobacteria, covering different niches. Careful analysis of the bacterial geosmin gene and gene tree suggests an ancient bacterial origin of the gene, that was probably successively lost in different time frames. The high sequence similarities in the cyanobacterial geosmin gene amidst freshwater and soil strains, reinforce the idea of an evolutionary history of geosmin, that is intimately connected to niche adaptation.

## Introduction

Cyanobacteria can be found in marine, brackish and freshwaters exhibiting both planktonic and benthic lifestyles, covering almost all ranges of temperatures and salinities, in symbiosis with a variety of organisms, in soil, caves, deserts, and buildings, and are among the oldest life forms that evolved throughout time^1,2^. It represents a prokaryotic group with huge diversity; in morphology presenting from unicellular to filamentous forms with specialized cells; chemo-diverse since they produce a panoply of bioactive compounds: antifungal^3,4^, anticancer^3,5^, antibacterial^3^, antiviral^5^, algicidal^6^, and antioxidant^7^, as well as toxic compounds noxious to the health of humans and wildlife^8,9^. Two of the most common volatile organic compounds produced by Cyanobacteria are 2- Methylisoborneol (MIB, PubChem CID:16913) and Geosmin (PubChem CID:29746)^10–12^. These compounds are frequently described in fresh and brackish waters Cyanobacteria, as they cause taste and odor problems in drinking water, fish and shellfish^11,13,14^. Although non-toxic to humans, these compounds change the perception of safety by the public, causing major economic losses in both sectors^10,11,14^.

Geosmin is greatly associated with terrestrial environments. It is assumed that the organisms responsible for the gross production of geosmin in soil are Bacteria belonging to actinomycetes - *Norcardia cummidelens; N. fluminea; Streptomyces Luridiscabiei; S. albidoflavus -* since they are typical soil inhabitants^15,16^. Other known producers of geosmin are fungi - *Penicillium discolour*, *P. crustosum*, *P. expansum*, *Botrytis cinerea*, and *Chaetomium* sp. - that are also present in soil and associated with fruit and vegetable spoilage^14,15,17^. Geosmin is also produced by higher plants, such as red beet^18^ and cactus flowers^19^ and is thought to be implicated in defense mechanisms, pollination, and competition^18–22^.

The species of Cyanobacteria producing geosmin are diverse, belonging to several distinct taxons, namely, multicellular strains of Oscillatoriales, Nostocales and Synechococcales^10,11,23^ and recently the unicellular Synechococcales *Coelosphaerium* sp.^13^, debunking the idea that only filamentous Cyanobacteria could produce geosmin^24,25^. Most of the knowledge on cyanobacterial producers is from freshwaters, probably due to the blooms related to odor and musty taste issues, while terrestrial, brackish and marine producers are rarely described or absent^11,15,24,25^. In freshwaters, the most frequent reported producers are the Nostocales (*Dolichospermum* and *Aphanizomenon)* and the Oscillatoriales (*Phormidium* and *Tychonema*)^10,11,25^ and in the soil, Nostocales of the genus *Nostoc* in symbiotic associations with cycads, lichens, mosses, and liverworts^24,26^.

Animals are not indifferent to geosmin, presenting behaviors of both attraction and repulsion towards it. The geosmin odor may represent the presence of freshwater, as it happens with camels and eels^27–29^ or may be synonymous of inedibility and presence of noxious microorganisms for fruit flies and humans^29^. In fact, humans smell and taste geosmin at very low concentrations and this compound has always received attention from various sectors: public health, academia, industrial and commercial^11,14^. Geosmin is recognized for the pleasant earthy smell in the air, especially when it rains and can give a freshness note to a perfume. However, when associated with water, food, and beverages the pleasant odor becomes an unpleasant earthy or musty taste^14^.

The molecular basis of geosmin production is geosmin synthase gene (*geo*A) that encodes for a bi-functional domain enzyme, with both the N and the C-terminal parts containing two metal-binding motifs typical of sesquiterpene synthases. These two motifs require Mg^2+^ as the catalyst for the formation of complex terpenoid molecules like geosmin^30^, and are responsible for the cooperative binding of the three catalyst metal ions, as well as the positioning of the substrate molecule for the cyclization reactions cascade that follows^31,32^. The protein domains share significant similarities and contain typical metal-binding motifs of class I terpenoid cyclases. The N-terminal part is responsible for the ionization and cyclization of farnesyl diphosphate (FPP) into germacradienol and inorganic pyrophosphate molecules, and the C-terminal part mediates the reactions of protonation, cyclization, and fragmentation of the precursor germacradienol molecules into geosmin and acetone^11,31,33,34^.

While mining for natural product gene clusters from cyanobacterial cultures, using a genome screening approach, we found a species producing geosmin. This finding pursued us to describe a novel Cyanobacteria species and explore the distribution and dispersion patterns of geosmin in Bacteria. In the present work, we describe the main genomic and morphological features of this organism and discuss the possible evolutionary scenarios for the geosmin synthase gene within Cyanobacteria and Bacteria, both in terrestrial and aquatic environments.

## Results

### Identification of secondary metabolite production potential through genome mining

The genome assembly of *Microcoleus asticus* sp. nov. was performed and its major statistical attributes are described in Table 1.

**Table 1:** Genome statistics of *Microcoleus asticus* sp. nov. Quality assessment and level of completeness of the genome assembly of *Microcoleus asticus* sp. nov. COGs - Clusters of Orthologous Groups of proteins, CRISPR - Clustered Regularly Interspaced Short Palindromic Repeats.

Attribute	Value	%
Genome size (bp)	7,502,480	100.0
DNA coding (bp)	6,017,946	80.2
DNA G + C (bp)	3,420,381	45.6
DNA contigs	296	—
Contigs N50	37,607	—
Longest contig	132,835	—
Total genes	7,648	100.0
Protein coding genes	6,349	83.0
RNA genes	68	0.9
Genes with function prediction	3,521	46.0
Genes assigned to COGs	5,349	69.9
Genes with signal peptides	636	8.3
Genes with transmembrane helices	1,280	16.7
CRISPR repeats	6	—

The quantitative assessment and annotation of the generated genome resulted in a value of 99% completeness (see supplementary Fig. S1) and the functional annotation of the predicted transcriptome is presented in supplementary Table S1.

**Figure 1:**
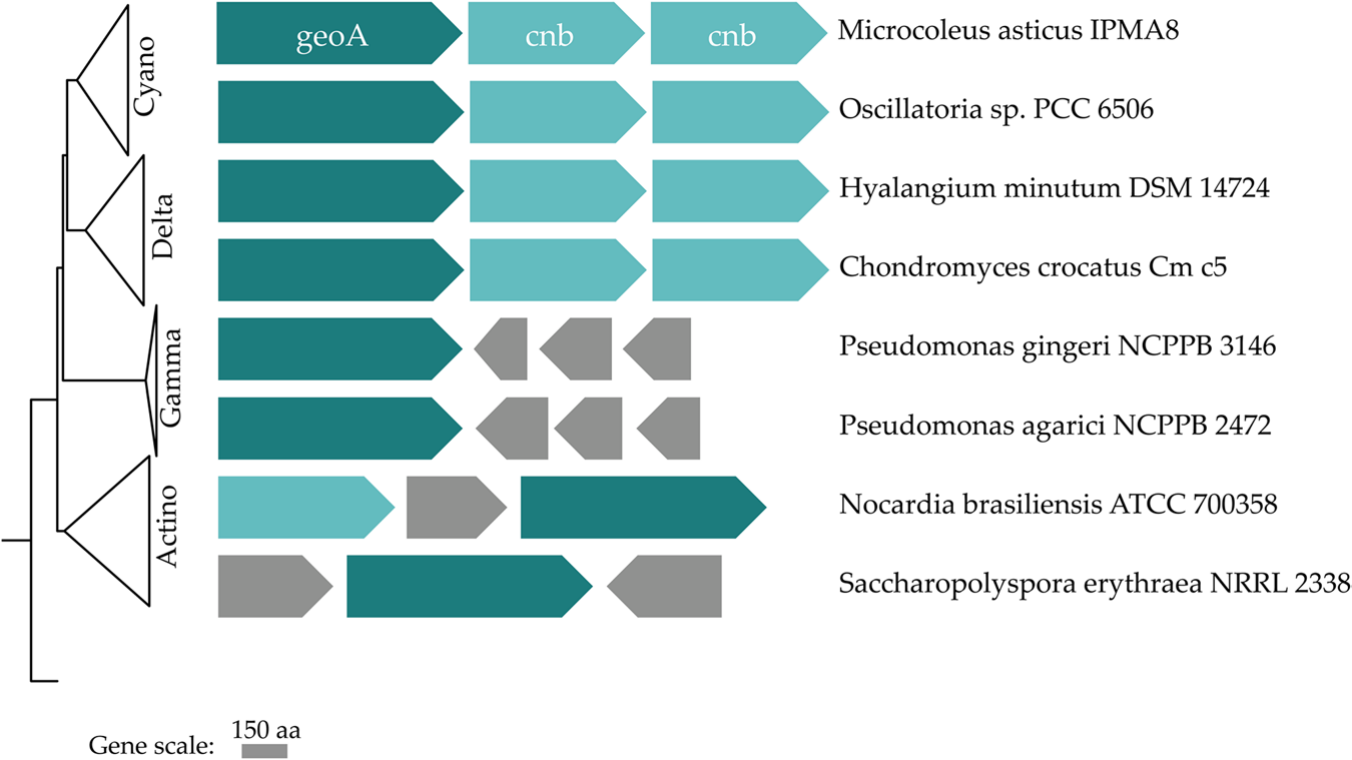
Schematics of the geosmin gene cluster and its synteny in *Microcoleus asticus* sp. nov. and the arrangement of genes related with geosmin synthesis identified in three distinct bacterial phyla: *Cyanobacteria* (Cyano), in the 2 classes of Proteobacteria: Deltaproteobacteria (Delta), Gammaproteobacteria (Gamma) and phylum Actinobacteria (Actino). Gene legend: *geoA* - geosmin synthase; *cnb* - cyclic nucleotide-binding protein; in grey are other genes.

After a genomic survey for non-ribosomal peptide synthase (NRPS), polyketide synthase (PKS), hybrid NRPS/PKS gene clusters and ribosomally synthesized and post-translationally modified peptides (RiPPs), we were able to identify the complete gene cluster for the synthesis of terpenoid geosmin, composed of the geosmin synthase gene (*geoA*) (758 aa in length), followed by two cyclic nucleotide-binding genes (*cnb*) (471 and 469 aa in length each) of the Crp/Fnr-type global transcription regulators (Fig. 1). Our organism presents the same cluster scheme as other Cyanobacteria and Deltaproteobacteria, where this cluster syntheny is preserved. Our *in silico* search for geosmin gene clusters in Bacteria, detected other cluster organizations in Gammaproteobacteria and Actinobacteria, as the examples presented in Fig. 1 demonstrate.

Other genes that participate in the synthesis of known cyanobacterial secondary metabolites were also detected, albeit, in none of them, the complete cluster was found. The incompletes gene clusters identified would code for: benzenodiol resorcinol, cyanopeptolin (*Dar*B was detected as well as genes with shared similarity), trichamide, and the mixed PKS/NRPSs nostophycin, nostopeptolide, and jamaicamide. Furthermore, we also identified the existence of two unknown terpene biosynthesis gene clusters showing the potential for alternative pathways for the synthesis of terpenoid compounds and other secondary metabolites that are still unknown.

### Analysis of terpenoid compound geosmin production by *Microcoleus asticus* sp. nov

**Figure 2:**
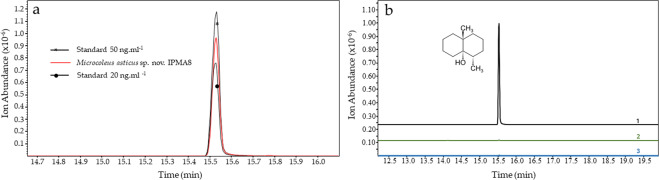
Analysis of the volatile metabolite geosmin produced by *Microcoleus asticus* sp. nov. using SPME GC-MS. Selected ion monitoring chromatogram in gas chromatography-mass spectrometry (GC/MS-SIM) of the ionic current of the selected geosmin ions (m/z = 111; 112; 125 and 126). (**a**) Geosmin detection in the *Microcoleus asticus* sp. nov. culture volatile extract; (**b**) Control analysis: 1- analytical geosmin standard (20 ng.ml^−1^), 2- no compound control, 3 - Z8 culture media.

The extract of volatile compounds produced by the isolate was analyzed to test if the geosmin complete gene cluster was, in fact, producing this terpenoid secondary metabolite. The odorous molecule geosmin was identified through headspace solid-phase micro-extraction (SPME). Figure 2 shows the ion monitoring chromatogram of the detection of geosmin from the volatile extract produced from *Microcoleus asticus* sp. nov., with retention time 15.5 min, matching the peaks for geosmin standards.

### Geosmin synthase gene is scattered throughout three bacterial phyla

**Figure 3:**
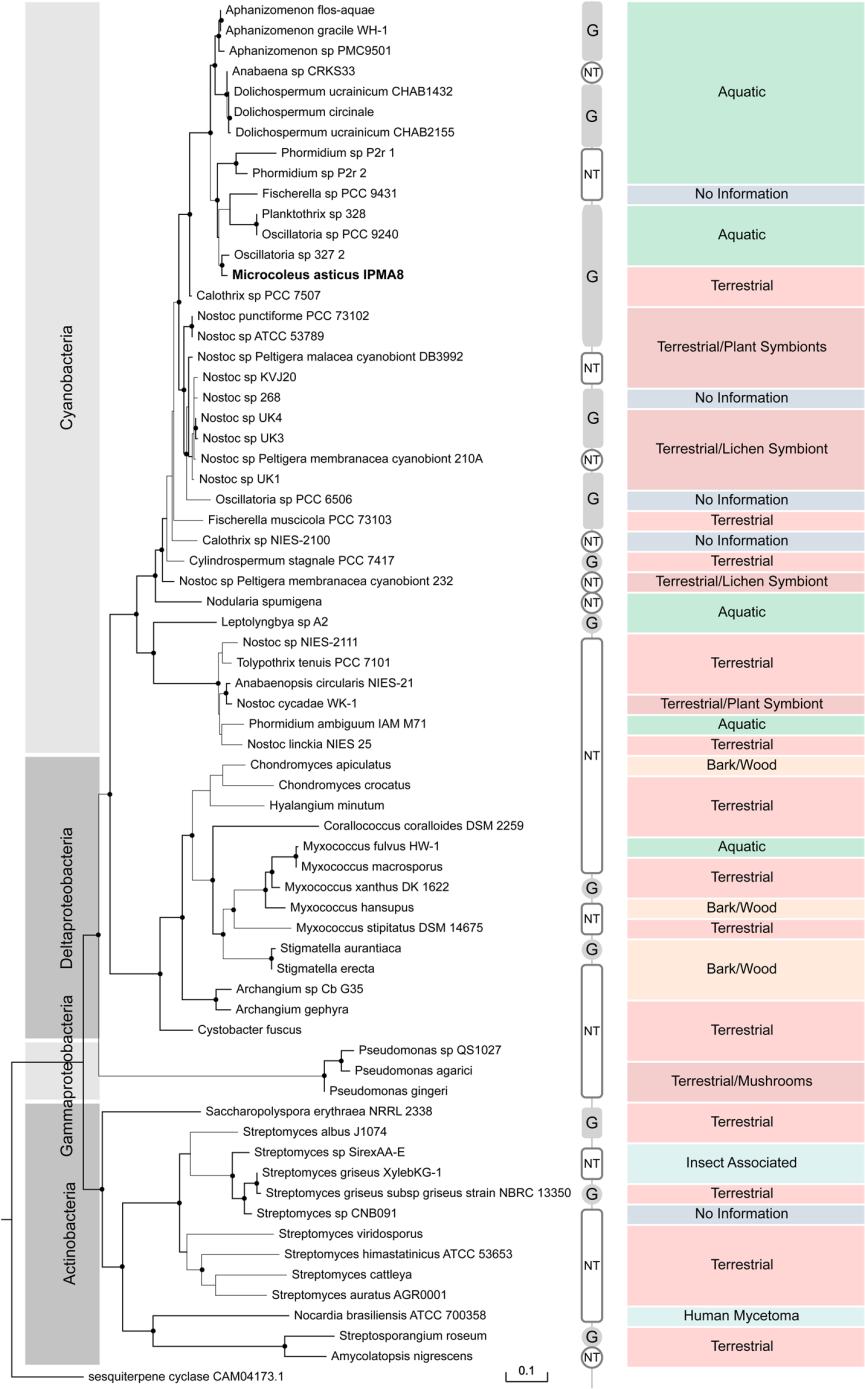
Phylogeny of the geosmin synthase gene in Bacteria calculated from a protein alignment of the geosmin synthase gene sequence. Tested geosmin producers are identified by the letter G and the letters NT identify strains that have not yet been analytically tested. The environmental origin of each strain is also shown. Black dots and thick branches represent maximum likelihood and posterior probability values higher than 85, respectively.

To better illustrate geosmin presence/production in Bacteria, it was fundamental to tackle different questions: How frequent is the gene spread in terms of phyla?; What species possess the genetic machinery to produce geosmin and even niche occupancy?; Can we trace the evolutionary history of the gene in Bacteria? To do so, we compiled a set of geosmin gene sequences, collected by BLAST searching public databases for complete and partial putative geosmin gene sequences. Our first outcome, was that sequences were restricted to only three bacterial phyla: Cyanobacteria, Actinobacteria, and Proteobacteria represented by two classes, Delta and Gammaproteobacteria. Besides being restricted, the geosmin synthase was not evenly distributed in those groups (Fig. 3), with just some few representatives of these groups presenting the gene.

The environmental sources of our set of bacterial strains are also depicted in Fig. 3, where it was possible to identify the occupancy throughout very diverse niches. Regarding Cyanobacteria, we identified 18 strains associated with terrestrial niches: 8 soil strains, 7 strains in symbiosis with lichens, 1 in symbiosis with liverwort and 2 strains that exist as symbionts of plant roots. The aquatic strains are mostly from freshwaters with only 1 out of 15 representatives being from brackish waters. Four strains do not have publicly available information, of niche occupancy. Deltaproteobacteria species are mostly associated with soil or decaying wood or tree bark and there is a single aquatic strain, the marine *Myxococcus fulvus*. Moreover, of the 3 Gammaproteobacteria strains, 2 are related to bacterial infections in edible mushrooms. Actinobacteria are mostly terrestrial strains, but 2 out of the 13 actinobacterial strains are associated with insects, while 1 was identified in a human lung infection. Figure 3 has also the indication of known geosmin producers, using information collected from the literature, and systematized in supplementary Table S2.

Taking into account the number of geosmin producers used in our phylogenetic study, we found that, of the 36 Cyanobacteria analyzed, 21 are known geosmin producers, whilst 2 out of the 14 Deltaproteobacteria strains have been tested positive regarding the synthesis of this metabolite. Furthermore, among the set of 13 Actinobacteria strains, there are 3 known producers of geosmin, while there is no information regarding the production of geosmin by the 3 Gammaproteobacteria strains in our dataset. In fact, to our knowledge, this is the first report of geosmin synthase gene in Gammaproteobacteria.

**Figure 4:**
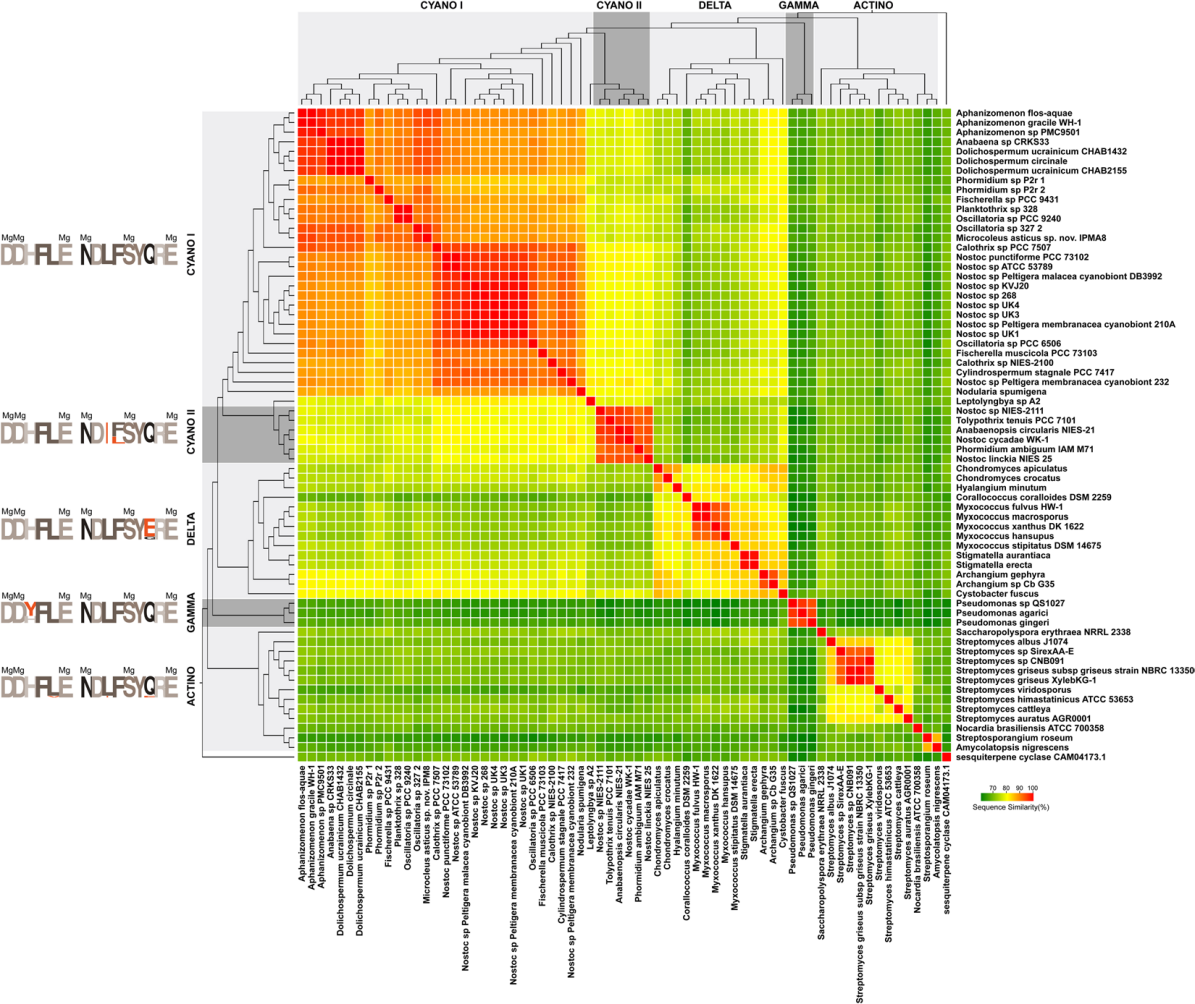
Analysis of sequence similarity of the geosmin synthase gene for the set of 66 bacterial strains. The Figure presents a logo representation of the amino acid residues of the two metal binding sites used to calculate the gene tree. The grey bars over the gene tree identify similar groups according to the amino acids of the two magnesium binding motifs. The heatmap is the graphical representation of a matrix of pairwise sequence similarities between all strains.

Overall, the gene tree topology matched the major phyla divisions of a 16S rRNA based species tree for Bacteria^35^. To better understand the evolutionary history of this gene in Bacteria, we compared the geosmin gene tree presented in Fig. 3 with a well-supported 16S rRNA based species tree^35^. Some differences in tree topology were visible between these two phylogenetic trees regarding the positioning of Cyanobacteria, Actinobacteria, Delta and Gammaproteobacteria. The geosmin synthase gene tree shows a close relationship between Cyanobacteria and Deltaproteobacteria, while in the species tree this closer relationship is not clearly visible. Nevertheless, Actinobacteria appears to be the most ancient phylum to harbor the geosmin gene as well as the most ancient of the four taxa divisions in the species tree. We decided to analyze these two phylogenies using a tree reconciliation algorithm to obtain support for a possible scenario for the evolutionary history of the geosmin gene in Bacteria (see Supplementary Fig. S2). Nevertheless, it did not reveal any clear patterns of evolution for the geosmin gene in Bacteria, probably due to a complex scenario involving several evolutionary drivers that probably lead to many geosmin gene loss events, which is the most dominant source of genetic variation in bacterial genomes^35,36^. The gene tree also exposed the formation of two sister clades in Cyanobacteria, with no apparent similarities in terms of genera or niche of the strains. Being the formation of the two groups in Cyanobacteria incongruent with its phylogeny and taxonomy and to complement the geosmin gene’s evolutionary history through Bacteria, we did a closer analysis of the two conserved magnesium binding motifs of the N-terminal half part of the geosmin synthase gene represented in Fig. 4, which is an important region for the catalysis reactions during geosmin synthesis. The N-terminal half part displays particular modifications between the groups and to better quantify the differences between these groups we performed a sequence similarity analysis of the gene sequence alignment, producing a similarity percentage matrix used to build the heatmap representation in Fig. 4. The differences and similarities between geosmin genes in Bacteria are noticeable, where the values of sequence similarity between all 66 bacterial strains of our dataset accentuate five major groups that share, within each group, similarity values between 76 to 100%. The two separate cyanobacterial groups can be distinguished, which we called Cyano I and Cyano II, as well as three groups we called Delta, Gamma, and Actino, and highlighted by the grey bars over the gene tree in Fig. 4. In Bacteria, the two motifs have three metal-binding residues each: the aspartate-rich motif has a universal consensus sequence DDXXX(D) and downstream of it, is the second motif, the NSE/DTE triad, with consensus sequence (N,D)D(L,I,V)X(S,T)XXXE^30,32,34,37^. The amino acid residues in bold are the metal ligands and are identified in Fig. 4 by the letters *Mg*. The five groups in the gene tree, share similarities in the two magnesium binding motifs which are represented by the amino acid logos in Fig. 4. We took as reference the Cyano I motif sequences DDHFLE and NDLFSYQRE, to highlight the residue substitutions in each group, which are colored in orange. These substitutions in both motifs, although do not appear to be critical to the binding capability, since it does not occur in the binding residues or in positions that are strictly conserved, can affect the motif conformation and the binding pocket with unforeseen consequences. Focusing on the magnesium binding motifs of the two cyanobacterial groups, the first motif of Cyano II group is identical to what is found in Cyano I, on the other hand in the NSE/DTE triad, there are two amino acid substitutions: the 3^rd^ residue (leucine) is replaced in Cyano II by isoleucine and the 4^th^ residue, a phenylalanine in Cyano I, is replaced by a leucine in 2 strains of Cyano II. Still, none of the strains in Cyano II were tested for geosmin production, so more tests should be made in order to clarify the impact of these amino acid modifications.

Cyano I group harbors cyanobacterial strains that share the same residue sequence of the Mg^2+^ binding motifs, but it is clear that in terms of the broader analyzed protein sequence *Leptolyngbya* sp. A2 is distinct from other cyanobacterial sequences, sharing with them 77 to 82% similarity, while strains in Cyano I share amongst them 83% to 100% similarity. Strains in Cyano II share high similarity values, 91 to 100%, and lower values with *Leptolyngbya* sp. A2 (78 to 79%). *Leptolyngbya* sp. A2, a MIB and geosmin producer from freshwater^23^ has its highest similarity values with the Nostocales strains in this study except for *Fischerella muscicola* and *Calothrix* sp. NIES-2100. The Cyano I group also reveals the existence of two clusters formed both by gene sequence and niche, where the freshwater strains in the clade *Aphanizomenon/Anabaena/Dolichospermum* share 94% to 100% sequence similarity and soil-related strains that include all *Calothrix* and *Nostoc* strains in our set, *Oscillatoria* sp. PCC 6506, *F. muscicola* and *Cylindrospermum stagnale* share amongst them 90% to 100% similarity. *Microcoleus asticus* sp. nov. IPMA8 lies in an intermediate group formed by strains from soil and aquatic origin: *Oscillatoria* sp. 327–2, *Oscillatoria* sp. PCC 9240, *Planktothrix* sp. 328 *Fischerella* sp. PCC 9431 and *Phormidium* sp. P2r, where the range of similarity values starts at lower values, from 87% to 100%.

Analysis of the geosmin gene tree highlights the probable close evolutionary history of geosmin synthase gene of Cyanobacteria with the Deltaproteobacteria gene, with higher similarity values shared with the two *Archangium* strains and *C. fuscus* (74 to 81%). In fact, the Deltaproteobacteria phylum shows a tight proximity among its gene sequences with similarity values ranging from 76 to 100%. Regarding Actinobacteria, the *Streptomyces* sp. clade forms a closely related group with clear differences in the protein sequence to the other actinobacterial strains, despite their similarities of the geosmin synthase metal-binding motifs. The *Pseudomonas* strains (*P. gingeri*, *Pseudomonas* sp. QS1027 and *P. agarici*), which we identified by *in silico* search, have low similarity values with all sequences from the other bacterial phyla, with the lowest similarity values (61%) with Deltaproteobacteria’s strains *M. xanthus* DK 1622 and *C. coralloides* DSM 2259 and Actinobacteria’s *S. roseum* and *S. cattleya*. The best similarity values with the *Pseudomonas* strains were identified for Deltaproteobacteria *C. fuscus* (67 to 70%) and Cyanobacteria *Fischerella sp*. PCC 9431 (69 to 70%). Taken as reference the Cyano I group, Actino is the group with more modifications which in turn occur with low frequency (L by V in the first site and L by I and Q by E in the second binding site), while Cyano II group has two modifications both in the second binding site: a complete replacement of L with I and F with L. Delta and Gamma groups have both one amino acid replacement: E by Q in the second binding site and Y/H in the first binding site, respectively.

### The selection pressures on geosmin gene in Bacteria

We quantified the selection pressures on geosmin gene using GARD algorithm to identify recombination breakpoints, which could increase the false points of positive selection pressure. This pre-analysis identified one recombination point in the amino-acid alignment. We then tested three different algorithms; SLAC, FUBAR and MEME and in all of them the global value of dN/dS is significantly higher than 1. We identified most codon sites as positive selection sites, indicative of strong diversifying selection pressure of the geosmin gene in Bacteria. The SLAC analysis points to pervasive (refereeing to the whole phylogeny) positive selection in 28 out of 269 sites and negative selection in solely 1 site (p < 0,05) while the FUBAR test also points to positive (35 sites) over purifying (21 sites) pervasive selection (pp > 0,9). The MEME algorithm, used for the identification of adaptive evolution in individual sites, shows positive selection in 55 sites (p < 0,05), confirming our hypothesis that several sites in the geosmin gene were subjected to positive or diversifying selection pressure.

### Morphological description of *Microcoleus asticus* sp. nov

**Figure 5:**
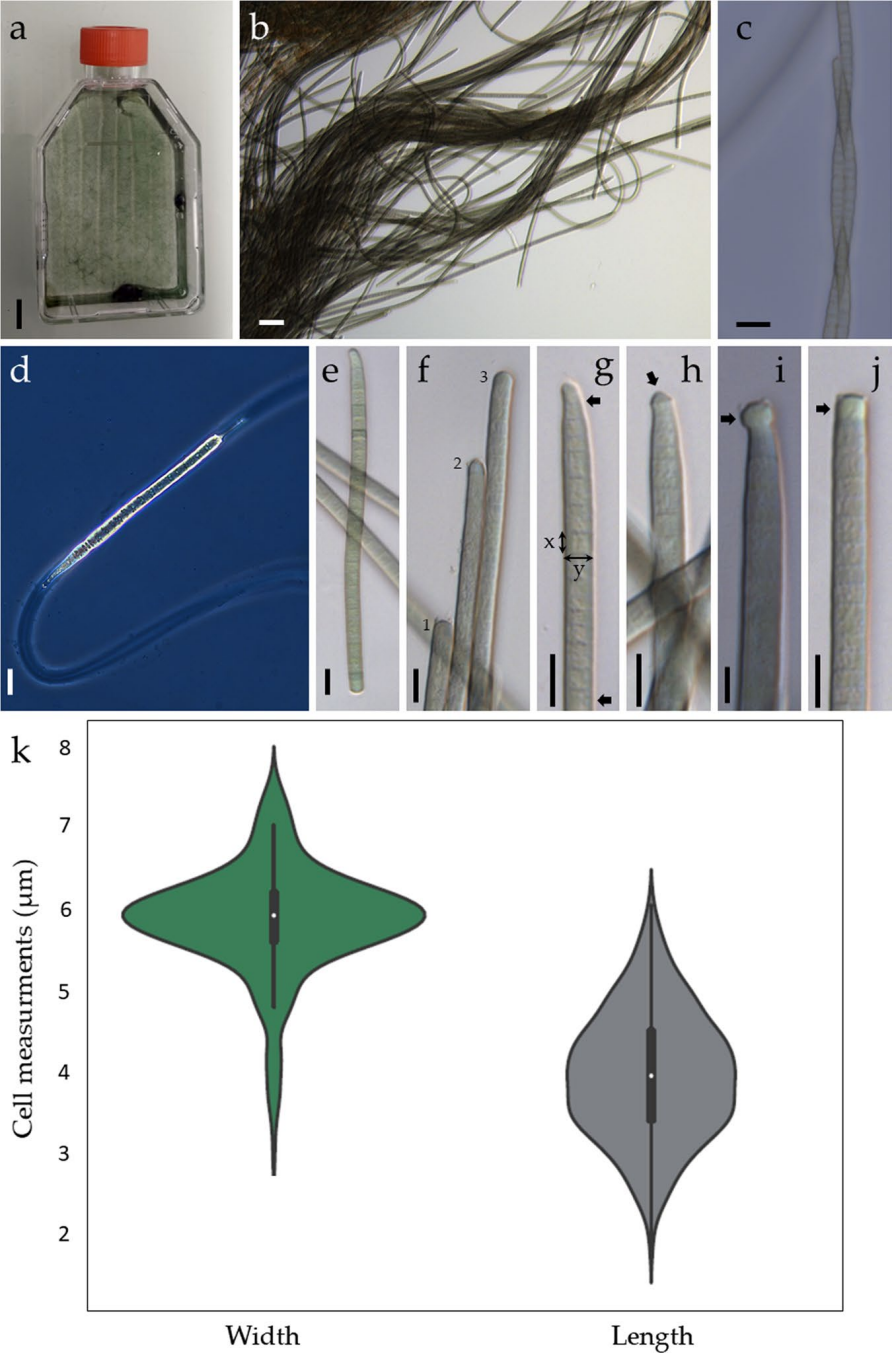
Morphology and morphometry of *Microcoleus asticus* sp. nov.: (**a**) mat in culture conditions (scale bar 1 cm); (**b**) filaments in low magnification; (**c**) entangled filaments; (**d**) surrounding sheath in phase contrast; (**e**) heteropolar filament; (**f**) variation in end cell morphology in straight filaments: 1 – truncated, 2 – conical, 3 – broadly rounded; (**g**) tapered filament, note a reduction of 64% in cell width towards the end between the two arrows, the lines represents the cell morphometric measurements, x - length, y – width; (**h**) tapered filament with a calyptra (arrow); (**i,j**) filaments with spherical and square hyaline membranous structures (arrows); (**k**) distribution density of the cells widths and lengths, the white dot indicates the median and the black bar represents the interquartile range, the black line represents the 95% confidence intervals after 500 measurements. Scale bars 10 µm.

The novel specimen is a terrestrial, free-living filamentous Cyanobacteria without heterocysts and akinetes. It forms dense mats in culture conditions in both liquid and solid media. In the liquid, the mat is both submerged attached to the bottom of the flasks and walls and also at the surface of the liquid forming aggregates with pockets of air (Fig. 5a). In solid, it dwells the medium and it grows in all its thickness. The filaments are dark green in color, uniseriate, straight, without false branching (Fig. 5b). Mucilage is present and visible in light microscopy; each filament is enwrapped by a single sheath (Fig. 5b). In natural samples the filaments were solitary but in dense cultures entangled filaments were visible with no evidence of a shared sheath (Fig. 5c). The filaments have movement capability and exhibit phototaxis. Cells are wider than long and rarely isodiametric, 5.87 ± 0.674 µm (CV = 11.4%) wide and 3.95 ± 0.776 µm (CV = 19.7%) long. The morphometry with the distribution density of the cell widths and lengths is presented in Fig. 5k. The minimum cell width measured was 2.90 µm and the maximum 7.62 µm, cell length had a minimum of 1.82 µm and a maximum of 6.05 µm. Filaments are cylindrical, heteropolar, with both straight and narrow ends (Fig. 5e). Tapered filaments can have a reduction of 64% in cell width towards the end (Fig. 5g). The terminal cells can have several morphologies: can be pointed in tapered filaments with or without a thickened membrane (calyptra) (Fig. 5g,h), rounded in strait non-calyptrate filaments (Fig. 5f) and can also present spherical, cylindrical or square hyaline membranous structures (Fig. 5i,j).

**Figure 6:**
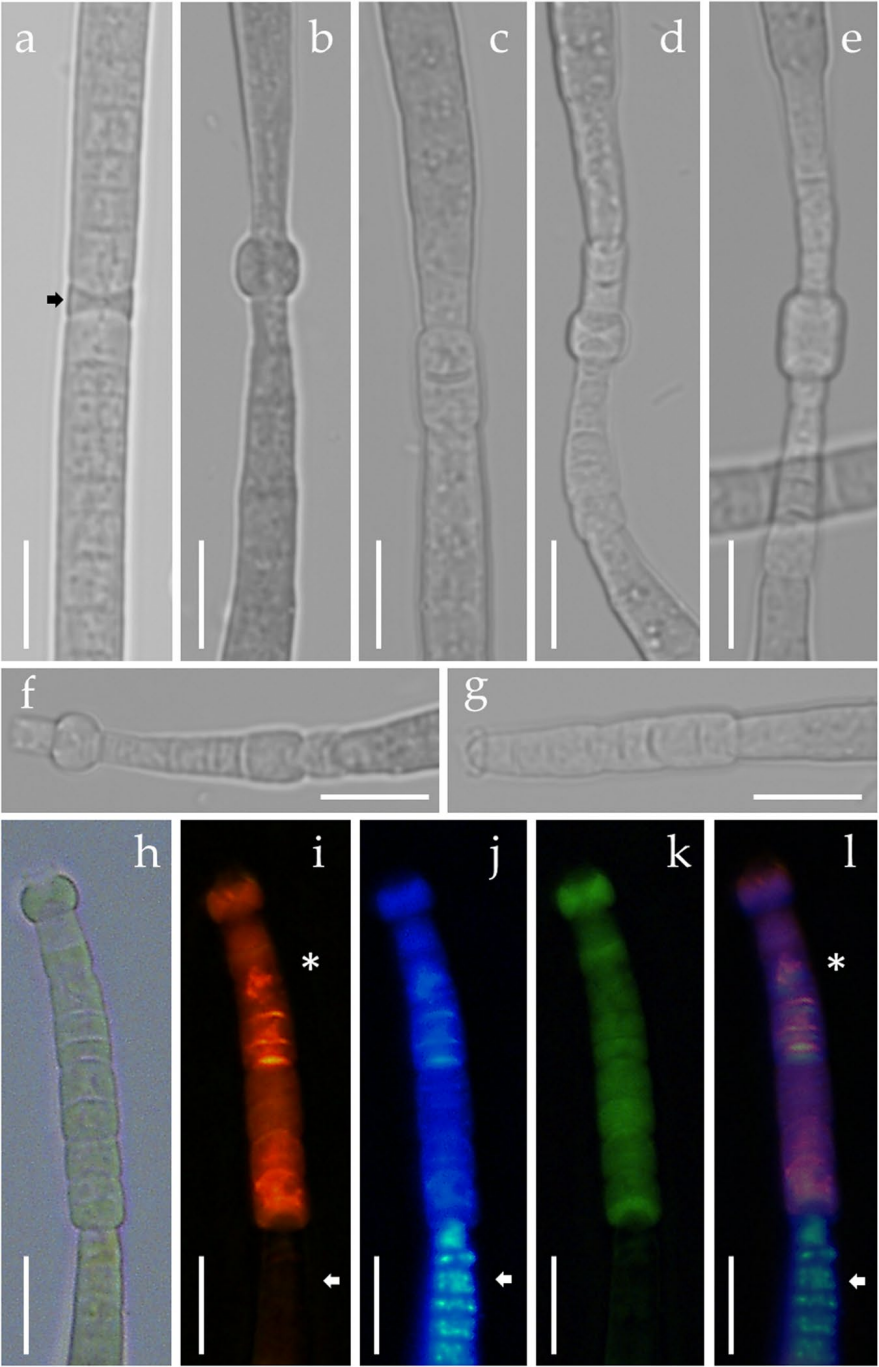
Necridia cells in *Microcoleus asticus* sp. nov.: (**a**) dying biconcave isolated cell (arrow); (**b,c**) dying cell in the form of a swollen nodule, can be circular or square; (**d,e**) group of dying cells around the nodule; (**f,g**) filaments with rests of dead cells still attached; (**h–l**) cells stained with cell viability imaging reagents for DNA and membrane integrity in fluorescence microscopy; (**h**) filament in bright field; (**i**) positive propidium iodide DNA staining of cells with compromised plasma membrane integrity (asterisk), negative propidium staining (arrow), RHOD filter; (**j**) NucBlue® live reagent DNA staining the nuclei of all the cells, DAPI filter, note the differential organized DNA fluorescence in live cells (arrow); (**k**) dead cell membranes green autofluorescence with blue light excitation, FITC filter; (**l**) composed image evidencing dead cells (asterisk) and live cells (arrow) in the same filament. Scale bar 10 µm.

Filament separation is by means of necridia and two types of these cells are present in this species (Fig. 6). One type is formed by only one dying cell in which the result filaments are straight, small and with round ends (hormogonia) that slide away from each other inside the sheath (Fig. 6a). The other type involves several dying cells in a very specific pattern: first a swollen hyaline cell is formed in the filament (Fig. 6b,c), after that, the cells around that hyaline nodule start to degrade (Fig. 6d,e) and the filament can break at any point along that extension of the degrading cells (Fig. 6f,g). The resulting filaments present tapered ends. The dying group of cells can be observed in fluorescence microscopy with cell viability imaging reagents (Fig. 6h–l). In Fig. 6h is the filament in bright field microscopy after the separation of necridia. Observation in bright field can be misleading since it can be interpreted as a different morphologic filament end. However, when stained with propidium iodide (Fig. 6i) and NucBlue® (Fig. 6j) it is visible the non-viable cells versus viable cells (Fig. 6l).

**Figure 7:**
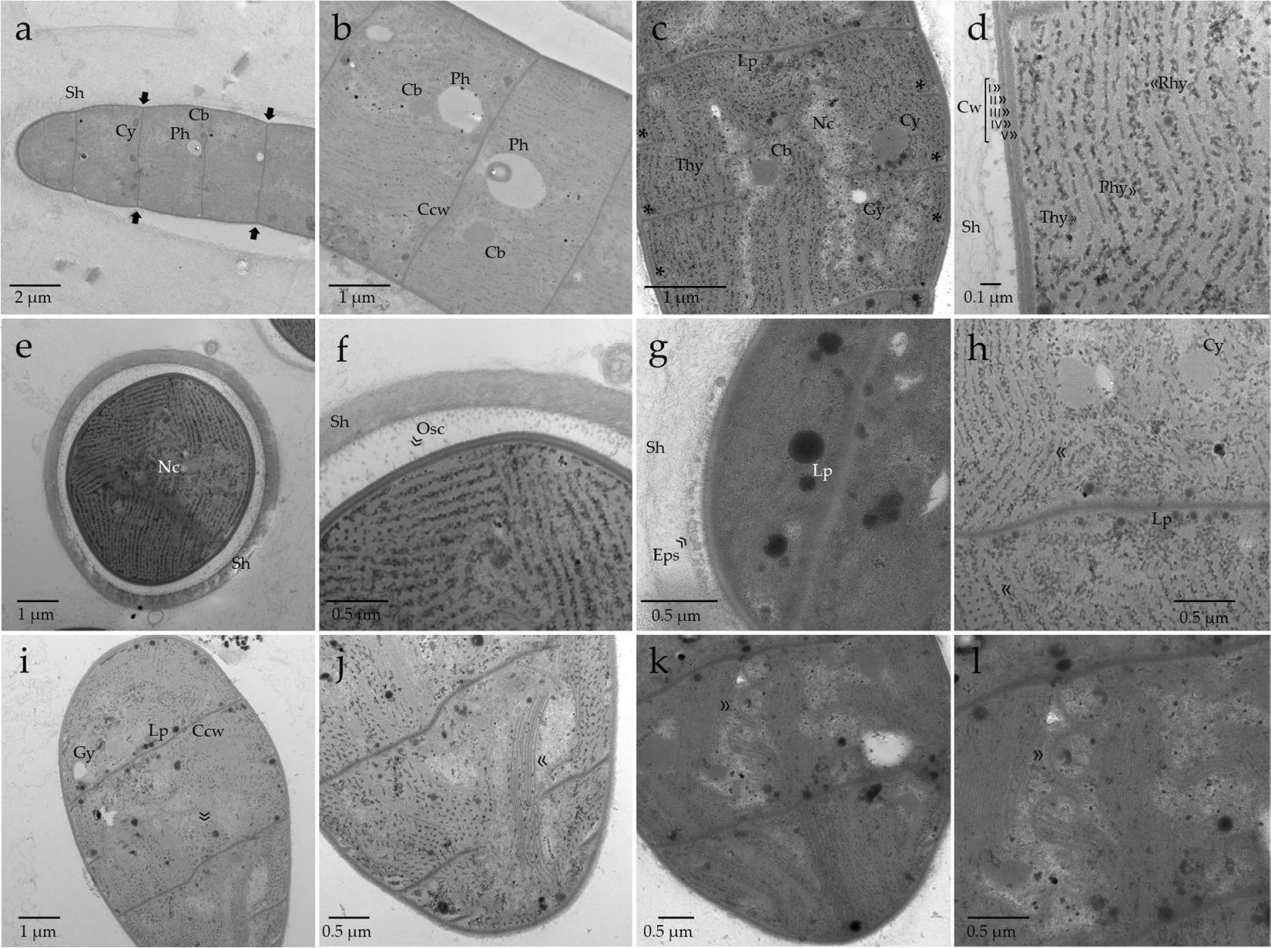
Transmission electron microscopy (TEM) micrographs of the cell ultrastructure. (**a**) General view of a longitudinal section of a filament at low magnification, note the constrictions at cross cell walls - arrows; (**b**) Close-up of a longitudinal section evidencing typical cell inclusions of Cyanobacteria cells; (**c**) Dividing cells, note the different stages in the development of the cell wall invaginations - asterisks; (**d**) Detail of cell wall (Cw) and thylakoid membranes (Thy): I ≫ S-layer, II ≫ outer membrane, III ≫ periplasmatic space, IV ≫ peptidoglycan layer, V ≫ inner cytoplasmatic membrane^106^, between the thylakoids are numerous phycobilisomes (Phy) and ribosomes (Rhy); (**e**) Cell in cross-section where is visible the central nucleoplasmic region (Nc), the arrays of thylakoids and the mucilaginous sheath (Sh); (**f**) Close-up of the cell wall and the sheath (Sh) in cross-section, note the Oscillin fibrils (Osc) attached to the s-layer^106,107^; (**g**) Detail of the mucilaginous sheath with excreted exopolysaccharides (Eps); (**h**) fasciculate thylakoid membranes in longitudinal section, note the different directions of the membranes some were cut longitudinally and others were cut transversely - arrows; (**i**) Oblique section of the filaments, note the lipid droplets near the cross-walls and the cylindrical membrane structure that resembles “thylakoid centers” – arrow^48,49^; (**j**) Close-up of the oblique section evidencing the curved fascicules of thylakoids (arrow); (**k**) spherical formations of the thylakoid membranes - arrow; (**l**) spherical formations of the thylakoid membranes in high magnification - arrow. Lp – lipid droplet, Cb – carboxysomes/polyhedral bodies, Ph – polyphosphate granules, Cy – cyanophycin granules, Gy – electroyaline storage granule (glycogen granule), Ccw – cross-walls, Cw – Cell wall, Nc – nucleoplasmic region with DNA and ribosomes, Thy – thylakoid membranes, Rhy – Ribosomes, Phy – phycobilisomes, Sh – mucilaginous sheath.

In cell ultrastructure it is visible the constrictions at cross-cell walls (Fig. 7a) and regular cyanobacterial cell inclusions, such as polyhedral bodies, polyphosphate granules and cyanophycin granules (Fig. 7a,b). Gas vacuoles are absent in this species. Cell division is in one plane and perpendicular to the cell wall, membrane invaginations for the new cells are visible at several stages of development simultaneously (Fig. 7c). The cell wall structure is gram-negative, formed by the S-layer, outer membrane, periplasmatic space, peptidoglycan layer and inner cytoplasmatic membrane (Fig. 7d). In the cell cross-section there is a well-developed mucilaginous sheath involving only one filament (Fig. 7e). In high magnification, we can see the oscillin fibrils attached to the s-layer that help the movement of the filament inside the sheath (Fig. 7f) and the excreted exopolysaccharides (Fig. 7g). Lipids and cyanophycin granules are located mainly at cross-cell walls (Fig. 7a,c,g–i). The nucleoplasmatic region is in the center of the cell (Fig. 7c,e). The thylakoids have a fasciculate arrangement of irregularly distributed and omnidirectional membranes (Fig. 7c–f,h–l). The fascicules run in parallel and form curves (Fig. 7i,j) to fully spherical formations (Fig. 7i,k,l).

### Phylogenetic analysis *Microcoleus asticus* sp. nov

**Figure 8:**
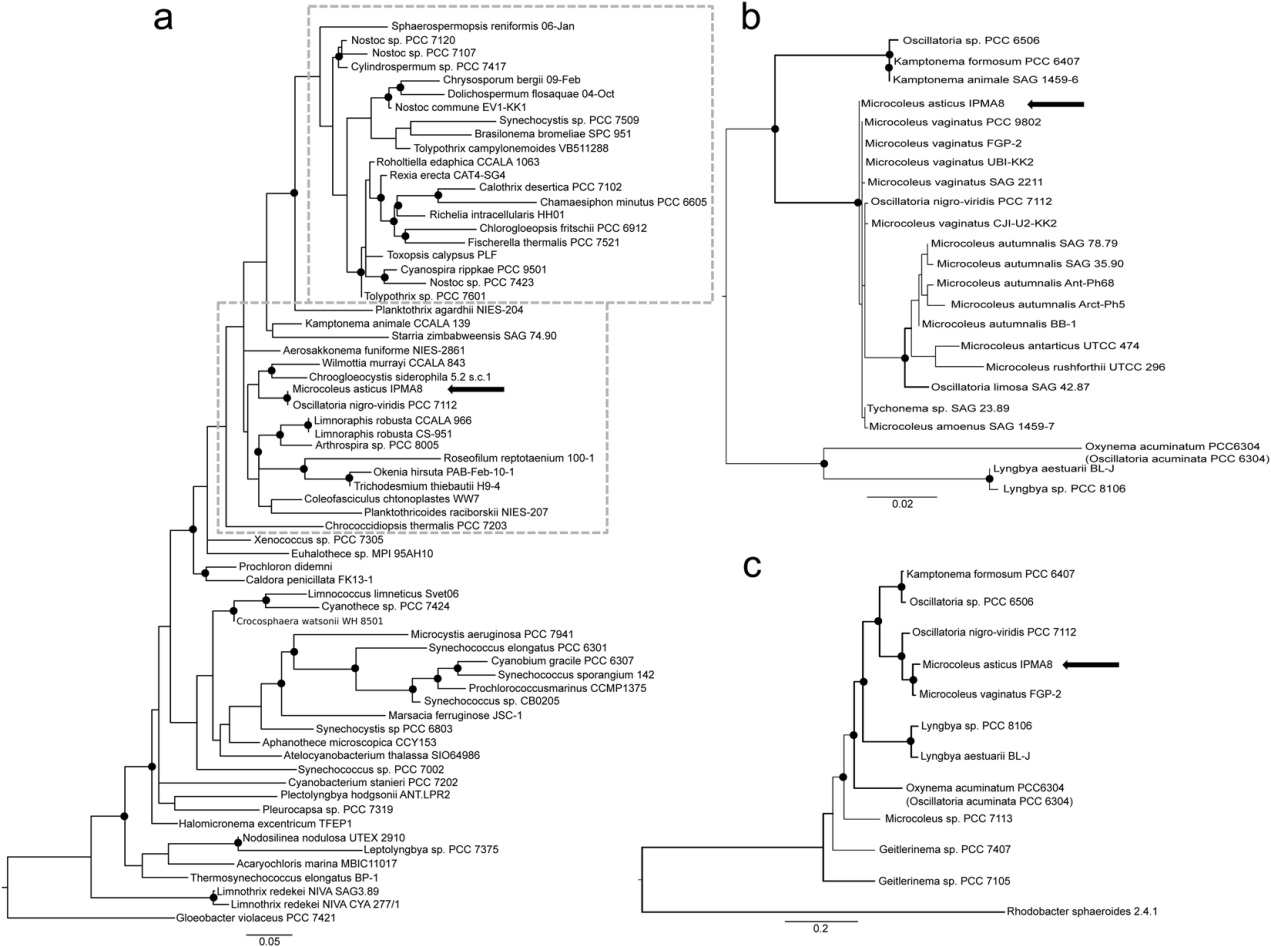
Phylogeny of *Microcoleus asticus* sp. nov. soil strain in the Cyanobacteria phylum. (**a**) Represents the identification of *Microcoleus asticus* sp. nov. as an Oscillatoriales strain among a set of 118 cyanobacterial strains^90^ where taxonomic orders of known geosmin producers (Nostocales and Oscillatoriales) in Cyanobacteria are highlighted by the grey dashed boxes. Values of Maximum Likelihood (ML) higher than 60 are represented by the black dots. (**b**) Presents 16S rRNA gene (1256 bp) refined placement of *Microcoleus asticus* sp. nov. among 23 strains of Oscillatoriaceae. ML and NJ values above 90 are represented by black dots and thicker branches, respectively. (**c**) Depicts a phylogenetic analysis using a set of 64 genes of 11 Oscillatoriales strains. Values of ML and Posterior Probabilities (PP) higher than 90 are represented by black dots and thick branch lines, respectively.

To perceive the phylogenetic context of this soil isolate, it is required to find its kindred strains. Our primary 16S rRNA based phylogenetic analysis (Fig. 8a) using a curated database of Cyanobacteria 16S rRNA, confirmed the isolate as a member of the order Oscillatoriales and a close relative to *Oscillatoria nigro-viridis* PCC 7112. In an effort to refine and validate this primary identification, we performed a close-up phylogenetic analysis, using the 16S rRNA gene, widely represented in genomic databases, which confirmed a close phylogenetic relationship of the isolate with other strains from the family Microcoleaceae (Fig. 8b). Finally, a refined phylogenetic analysis of a set of 64 housekeeping genes (see Supplementary Table S3) present in 11 fully sequenced Oscillatoriales strains, to unveil its relationship with other strains from the *Microcoleus vaginatus*/*Microcoleus autumnalis* (former *Phormidium autumnale*) clade, as presented in Fig. 8c. Our phylogenetic analysis supports this isolate as new species, being the closest completely sequenced genomes the *Microcoleus vaginatus* FGP-2 and *Oscillatoria nigro-viridis* PCC 7112. The similarity matrix constructed from the set of 64 genes of these three genomes shows differences of 3% with *M. vaginatus* FGP-2 and 7% with *O. nigro-viridis* PCC 7112 (supplementary Table S4). We further compare the average nucleotide identity (ANI), the differences in G + C content and *in silico* DNA–DNA hybridization (DDH) between our isolate and its closest relative *Microcoleus vaginatus* FGP-2. For the ANI^38^ analysis results showed that average nucleotide identity is91.71% (Table 2). The DDH estimate^39^ (GLM-based) was 46.40% with a distance of 0.0805 with a probability that DDH > 70% (same species) of 10.65% and probability that DDH > 79% (same subspecies) of 2.28% and a difference in % G + C of 0.44.

**Table 2:** Average nucleotide identity (ANI) values for *Microcoleus asticus* sp. nov. and *Microcoleus vaginatus* FGP-2^38^.

Metric	Value
OrthoANIu value (%)	91.71
Genome *M. asticus* length (bp)	7,351,140
Genome *M. vaginatus* length (bp)	6,678,960
Average aligned length (bp)	3,655,706
Genome *M. asticus* coverage (%)	49.73
Genome *M. vaginatus* coverage (%)	54.73

### Taxonomic description

The description was made under the provisions of the International Code of Nomenclature for algae, fungi and plants (Shenzhen Code, 2018 ed.)^40^. *Microcoleus asticus* C. Churro, A.P. Semedo-Aguiar, R.B. Leite sp. nov.

Here designated

Etymology: of/located in a city, city, urban

Type Locality: Lisbon city center, urban area, Av. Infante Santo, coordinates: 38°42′33.7″N 9°10′00.3″W, Portugal.

Habitat: Cyanobacterial mat in soil dominated by *Nostoc* sp. in a street flowerbed from an urbanized area.

Description: Filaments form dark olive-green mats, only entangled when the densely mat is formed, otherwise, solitary, motile and enwrapped in a single sheath. The sheath is prominent, firm, colourless and hyaline. Filaments are cylindrical, heteropolar (straight and/or attenuated towards the ends) and constricted at the cross-walls. Apical cells can be broadly rounded to conical with or without calyptra. Cell content is granulated with the cell inclusions visible in light microscopy. Lipid droplets are located near the cross-walls, polyphosphate granules and polyhedral bodies are in the center of the cell and cyanophycin granules are mainly near the cross-walls, but can also be scattered in the cytoplasm. Gas vacuoles are absent. Cells are wider than long and rarely isodiametric; 2.90–7.62 (5.87) µm wide and 1.82–6.05 (3.95) µm long. Cell division is in one plane and several divisions occur simultaneously. Filament dispersal is by means of motile hormogonia or long filament breakage by the aid of necridia. Necridia can be formed by the degradation of only one cell or by a group of adjacent cells. The thylakoids have a fasciculate arrangement. The fascicules run in parallel and form curves to fully spherical formations. The genome data is available at DDBJ/EMBL/GenBank under the accession number: PRJNA531034. Phycobank registration http://phycobank.org/102090.

Diagnosis: The main distinctive morphological characters are the type of fasciculated thylakoid arrangement and the type of necridia cells.

Holotype: Held in the Herbarium of University of Coimbra (COI), Department of Life Sciences, University of Coimbra Coimbra, Portugal in a metabolically inactive state in the form of preserved material with the strain identifier COI00097100 and the information available online at: https://coicatalogue.uc.pt/index.php.

Isotypes: Cultures of *Microcoleus asticus* are held in in the Collection of Microalgae of University of Coimbra (ACOI) at the Department of Life Sciences, University of Coimbra, Coimbra, Portugal and at the Culture Collection of Cyanobacteria of the Portuguese Institute for Sea and Atmosphere, I. P. (IPMA, IP), Av. Alfredo Magalhães Ramalho, 6, 1495–165 Algés under the strain identifiers ACOI 3416 and IPMA8 respectively. Preserved isotypes are held in the Herbarium of University of Coimbra (COI), Department of Life Sciences, University of Coimbra Coimbra, Portugal with the strain identifiers COI00097101; COI00097102; COI00097103 and the information available online at: https://coicatalogue.uc.pt/index.php.

## Discussion

We have isolated and sequenced the genome of a soil Cyanobacteria that is able to produce geosmin. Based on a phylogenetic study and morphological analysis a new representative of the *Microcoleus vaginatus*/*Microcoleus autumnalis*^41,42^ clade was confirmed and named *Microcoleus asticus*. The strong support of our phylogenetic and the results of average nucleotide identity, and DNA-DNA hybridization, allowed us to confirm that it is a new species with close proximity to *Microcoleus vaginatus* FGP-2, a free-living isolate collected from a desert-soil crust^43^ and a member of the Microcoleaceae IV family ^41,44,45^. The ANI values were below the 95–96% similarity threshold^46,47^ and DDH estimate also below 70% similarity threshold for species boundaries^39,46,48^ indicating that our isolate is a different species from *M. vaginatus*. The main distinctive morphological characters are the type of fasciculated thylakoid arrangement and the type of necridia cells. The curved and spherical formations of the thylakoid membranes that resemble the centers for thylakoid connectivity described by Nevo *et al*.^48,49^ are not described for *Microcoleus*. Spherical thylakoid formations are present in several distinct Cyanobacteria^44,50^, but none of the reported combinations of spatial, directional, and morphological rearrangement of the thylakoids are similar to the ones present in *Microcoleus asticus* sp. nov^44,50^. Necridia cells are also different in *Microcoleus asticus* sp. nov., beside the separation disc formed by one cell that is typically described^44^, this species also presents fragmentation by a group of degrading cells. Surprisingly the resulting filaments have tapered ends. Tapered end cells is usually attributed to filament maturation rather than newly separated filaments in Oscillatoriales^44,51^. All the other morphological characters are congruent with the genus *Microcoleus sensu stricto* with the type species *Microcoleus vaginatus* (Vaucher) Gomont *ex* Gomont (1892). Nevertheless, morphology is quite similar within some groups of the order Oscillatoriales (ex. the genus *Phormidium*) so, genetic information is necessary for the distinction^41,42^. *Microcoleus* is worldwide distributed and mainly associated with soil, aerophytic and epiphytic habitats and is one of the main constituents of biological soil crusts^43,52–54^. In soil environments Cyanobacteria play an important ecological role providing a nitrogen source in symbiotic relationships and producing phytohormones required for plant growth and development^55,56^, in soil particle aggregation, erosion reduction, increasing water penetration and retention and nutrient recycling^57–60^. Although the knowledge in the diversity of soil Cyanobacteria is increasing, much is still unknown regarding species composition and bioactive compound production.

The *in silico* mining of the genomic data identified a complete geosmin synthesis gene cluster, and the analytical analysis for volatile compounds allowed us to conclude that *Microcoleus asticus* sp. nov. IPMA8 is actively producing geosmin.

Our primary search for the geosmin synthase gene in bacterial public genomic databases allowed the identification of this gene in Gammaproteobacteria - which to our knowledge, is reported here for the first time - as well as in Deltaproteobacteria, Actinobacteria, and Cyanobacteria. Our efforts to map the presence of the geosmin gene in Bacteria revealed a restricted distribution, to these three phyla, crossing distinct genera from different niches, suggesting a possible rich evolutionary history of the gene. In our analysis the congruence between the bacterial species tree and the gene tree confirms a probable ancient origin of geosmin gene in Bacteria, that might have resulted from a mixture of evolutionary processes difficult to disentangle but that we identified as dominated by gene loss.

It is noteworthy that even in Cyanobacteria the distribution of geosmin gene is not heterogeneous, from the eight orders described for this Phylum, the Oscillatoriales, Nostocales and Synechococcales harbor the majority of known geosmin producers^10,11,24,44^. The gene cluster in Cyanobacteria is a mostly conserved one; to our knowledge the only cyanobacterial strain for which a different cluster organization was detected is *Phormidium* sp. (Pr_1 and Pr_2)^61^. The apparent disparity of the Cyano II group when compared with the straightforward divisions in the species tree could be explained with minor specific alterations in taxon and niche, since geosmin genes studied here appear to come from a common ancient Cyanobacteria and thus are orthologous genes. The geosmin gene cluster has two global transcription regulator genes, known to modulate cellular signals associated with responses to environmental stress^62^. The high conservation of the arrangement of the cluster, in Cyanobacteria, could thus indicate its high importance in controlling environmental adaptation, which is supported by the results described in other studies, indicating that the synthesis of geosmin and other volatile organic compounds, could be related with defense/offense mechanisms towards other microorganisms^63,64^. In *Nostoc punctiforme* PCC 73102, formation of geosmin results from conversion of the sesquiterpene precursor farnesyl diphosphate. However, upon expression of the enzyme in *E. coli*, no geosmin is produced, but instead synthesis of alternative products of geosmin synthases, such as germacradienol, germacrene D, and germacrene A, were observed, suggesting a dependency for environmental factors or other enzymes and metabolites for the active production of geosmin^65^.

Cyanobacteria and Deltaproteobacteria have very conserved and similar gene architecture, with a three gene operon assembly, whilst in Gammaproteobacteria and Actinobacteria this construction is not widely conserved. When circumspect geosmin synthase sequences, we find that Actinobacteria representatives are the most distant phylogenetic relative to Cyanobacteria while Deltaproteobacteria’s protein sequences are the closest (see Fig. 3). Otherwise, when the motifs in these protein sequences are analyzed in detail, Actinobacteria has globally more similarities with Cyanobacteria, despite having more modified amino acid positions (see Fig. 4). Actinobacteria are major components of both soils and freshwaters^66^. So, the inferences made from these patterns of evolution cannot be made separately, since these are probably the result of distinct evolutionary events acting on these phyla either simultaneously or in different time frames.

Even though half of the strains in our cyanobacterial dataset are associated to soil related environments (18 out of 36), a considerable part (15), has an aquatic origin and only one is found in brackish waters. It is also evident that free living terrestrial Cyanobacteria that carry the cluster, including our specimen, are more common than previously reported. This implies that Cyanobacteria are likely important contributors for the geosmin production in soil. Cyanobacteria from marine environments with the genetic capability to synthesize geosmin were not identified in our survey, which is congruent to what has been described in other studies^11,15,25^. However, as stated by Jutner and Watson^25^ salinity by itself is not impeditive of geosmin production since Cyanobacteria from brackish and high salinity environments are able to produce it. Additionally, there is only one marine organism in our data set carrying the full geosmin gene cluster which is the Deltaproteobacteria *Myxococcus fulvus* HW-1^67^. *Myxococcus* are mostly associated with terrestrial environments and marine specimens have only recently been discovered^67,68^. From an evolutionary perspective the salinity barrier in habitat expansion can be difficult to overcome^66^. Nevertheless, according to Sánchez-Baracaldo 2015^69^ the origin of marine planktonic cyanobacteria lies in freshwaters and benthic marine ancestors. The brackish water *Nodularia spumigena* CCY9414, for example, evolved from freshwater relatives by the end of the Pre-Cambrian period^69^. This strain carries the geosmin gene (*geo*A accession Number: WP_063873980.1). So, there is the possibility that marine cyanobacterial geosmin producers are already present in marine environments or perhaps the geosmin gene had no specific advantage in the sea and was lost throughout time. Interestingly, in Cyanobacteria it is noticeable the high sequence similarity of the geosmin gene among strains from freshwater environments as well as higher values for strains from soil symbiotic related environments, reinforcing that the evolutionary history of this gene could be related to niche adaptation.

Some questions can be posed at this time: Could these similarities in the metal-binding motifs and the broader protein sequence imply that the geosmin gene was acquired by Cyanobacteria from Actinobacteria? But what about the Cyano II group, was it a later acquisition to the cyanobacterial phylum?

A congruence test of the geosmin gene tree with a well-supported Bacteria species tree revealed no evidence for any specific pattern of evolution, instead, it appears that selection is acting towards the elimination of genes that were probably representing excessive energy costs. Nevertheless, our scrutiny of the natural selection pressures on the geosmin gene, shows clear evidence of strong positive selection when tested for both whole bacterial phylogeny and codon sites. These results support our hypothesis of an early presence of the geosmin gene in the bacterial kingdom, which was successively lost in the most derived branches of the species tree. The fixation of beneficial mutations, from positive natural selection, ultimately leads to differences among species and adaptation to different niches. Many bacterial strains have kept the geosmin gene and the ability to synthesize the metabolite, as it is clear by the present-day producers in Actinobacteria, Deltaproteobacteria and Cyanobacteria. Recent studies describe that Cyanobacteria kept the geosmin gene through purifying selection^70^. Also in Cyanobacteria, others have proposed a similar evolutionary history for the cyanotoxin microcystin, synthesized by several genera, proposing that negative selection was the main driver for the sporadic distribution of microcystin synthesis in this phylum: the toxin was present in ancient cyanobacteria and was successively lost throughout the species tree, refuting the lateral gene transfer hypothesis for this gene cluster^71^. These two examples are specific of Cyanobacteria and of distinct genes, which have their on environmental queues, but show that distinct evolutionary processes could be pressuring and shaping bacterial genomes and particularly the geosmin profile in Bacteria. We identified only three *Pseudomonas* strains, with the geosmin gene, which we do not know if are effective geosmin producers. Interestingly, several studies on geosmin biodegradation have identified strains of *Pseudomonas*, *Sphingopyxis*, *Chryseobacterium*, *Sinorhizobium*, *Stenotrophomonas*, *Novosphingobium* (Gamma, Beta and Alphaproteobacteria, and Actinobacteria) with potential to be used in bioremediation of geosmin and 2-MIB in aquaculture and water treatment facilities, often in bacterial consortiums in specific niches^72–75^. Several bacteria evolved to rely on less abundant and less explored carbon sources as their primary carbon source like monoterpenes and other volatile organic compounds^76^, which could ultimately result in losing the ability to synthesize terpenoids like geosmin, in specific environments. How this gene was kept and/or lost by distinct members of Bacteria, especially in the Phyla we studied here, is a result of many evolutionary processes that acted simultaneously or in different time frames or in different environments and different microorganisms. Our data support that this gene, was present in an ancient bacterium, probably an ancestor of Actinobacteria, but due to a rich evolutionary history was successively lost by several groups of Bacteria, resulting in this patchy presence in the taxa discussed.

Current knowledge on geosmin distribution, points to a bacterial originated soil compound that is strongly precepted by animals and also endogenously produced by higher plants, where terpenoids have been linked to defense mechanisms^18,19,21,22,27,29^. Nevertheless, the exact function of this compound and the adaptive advantage in “retaining” this gene cluster in Bacteria is still unknown. It is clear the near absence of marine geosmin-producing organisms, which pushes us to pursue the cunning halotolerant geosmin gene carrier Cyanobacteria. In fact, our study demonstrates that bacterial, as well as cyanobacterial, soil representatives could still be identified, diversifying the origins of geosmin producers that could have biotechnological uses. Over the last years, the increasing number of reports of geosmin occurrence in freshwaters states them as significant contributors for the production of this organic volatile compound in this environment. Likely, the increasing reports on terrestrial Cyanobacteria producing geosmin, indicates that they are also key players of its production in soil. Our study demonstrates that geosmin in soil can have multiple cyanobacterial origins resulting from intrinsic factors and environmental drivers during evolution.

## Methods

### Sample collection, isolation and culture maintenance

The cyanobacterial specimen used in this study was obtained from soil mats of the Cyanobacteria *Nostoc* sp. from an urbanized area in Lisbon, Portugal. A sample of the *Nostoc* sp. mat was collected and washed with Z8 culture medium. The washings were observed under the inverted microscope Leica® DMi8 and cyanobacterial filaments were isolated by micromanipulation with a glass capillary. Only one filament was picked up at each time, washed in new culture medium several times and placed in a culture flask to propagate. Successfully established cultures were maintained at 20 ± 1 °C with a light intensity of 10 μmol·photons·m^−2^·s^−1^ and a 12:12 h light: dark cycle.

### Morphological characterization

The specimen was studied using both optical and electron microscopy. The morphological characters evaluated were: filament color and shape, cell dimensions (length and width), constrictions at the cross-cell walls, shape of apical cells, presence/absence of sheath, false branching, calyptra and necridia^44^. The ultrastructure aspects analyzed were: arrangement and vacuolization of the thylakoids, type of cell division and cellular inclusions^44^. Captures were taken with a Leica® DFC7000T digital camera using a Leica® DMi8 inverted fluorescent microscope at 1000× magnification. The cell measurements were performed using LAS V4.12.0 Leica® software 2017, at least 500 measurements were done in 100 different filaments. The description and identification of the Cyanobacteria specimen was done based on the following manuals and bibliography^40–42,44,51,77^.

Cell viability was accessed in live cells using the ReadyProbes™ Cell Viability Imaging Kit (Blue/Red) from Invitrogen™ composed of NucBlue® Live reagent and propidium iodide following the manufacturer’s instructions. The cells were visualized under the Leica® DMI8 inverted fluorescent microscope and photographs were taken with a Leica® DFC7000T camera.

The cell ultrastructure was analyzed with transmission electron microscopy following the method describe in Churro *et al*.^78^ with minor modifications. Briefly, cell material was prefixed in 2.5% glutaraldehyde in Z8 culture medium for 30 min at room temperature and then transferred to 2% paraformaldehyde and 2.5% glutaraldehyde in 0.1 M cacodylate buffer overnight at 4 °C. Cell material was washed three times with 0.1 M cacodylate buffer and immobilized in 2% low melting point agarose. Post-fixation was in 1% osmium tetroxide, 1.5% potassium ferrocyanide in 0.1 M cacodylate buffer for 3 h on the ice and protected from light. Cells were then washed three times in 0.1 M cacodylate buffer for 10 min. To increase cell contrast, cells were incubated with 0.5% uranyl acetate for 1 h in the dark at room temperature and rinsed with distilled water for 15 min. Cells were dehydrated in a graded ethanol series (30, 50, 70, 95%, 20 min each, and absolute ethanol 3 × 20 min) at 4 °C, with a final step of propylene oxide for 10 min. After dehydration, cells were embedded in Spurr’s low-viscosity epoxy resin overnight, placed in flat embedding silicone rubber molds and polymerized at 60 °C for 24 h. Sections were post-stained with 2% uranyl acetate (5 min in the dark) and lead citrate (5 min). Sections were examined with a Hitachi® H-7650 transmission electron microscope.

### DNA extraction and sequencing

The DNA was extracted from the cultures using the DNeasy Plant Mini Kit, Quiagen®. The total DNA concentration was quantified using the Qubit™ Fluorometric Quantitation, Thermo Fisher Scientific®. The DNA libraries were created using Nextera DNA Library Preparation Kit, the purified DNA was sequenced on an Illumina NextSeq. 500 (Illumina, San Diego, CA, USA) and the sequencing was executed by Instituto Gulbenkian de Ciência at the Genomics Facility in Oeiras, Portugal. Genome assembly procedures were done as we have previously described for other genomic studies^79^.

### Genomic analysis, assembly and general features

The genome was assembled using SPAdes v3.6.10^80^. We used QUAST^81^ to calculate the quality statistics of the genome assemblies. Annotation of the genome was done with PROKKA v1.11^82^ and RAST v2.0^83^ using default parameters to identify putative genes (coding and non-coding sequences). Identification of orthologs proteins clusters was accomplished with eggNOG v4.5^84^ and the identification of CRISPR repeats, typical in Cyanobacteria, was performed with CRISPR Recognition Tool CRT v1.1^85^, considering a minimum of 3 repeat units. The prediction of transmembrane topology and signal peptide sites was done using Phobius^86^. To evaluate the presence and possible origin of prophage sequences, identification and annotation of these sequences were performed using PHASTER^87^. The quantitative assessment and annotation completeness of the assembled genome was performed with BUSCO v3^88^, by comparison with BUSCO’s orthologues database set for Cyanobacteria, odb9. We used the online tool antiSMASH v4.0^89^ to identify the presence of non-ribosomal peptide synthase (NRPS), polyketide synthase (PKS), hybrid NRPS/PKS gene clusters and other domains of secondary metabolites produced by Cyanobacteria.

### Phylogenetic analysis

In order to address the phylogenetic relationships of *Microcoleus asticus* sp. nov. IPMA8 in the cyanobacterial phylum a phylogenetic tree of the 16S rRNA gene sequences of the 118 strains from a public dataset of cyanobacterial 16S rRNA genes collected in CyanoType v.1 database^90^. This is a curated database of 16S rRNA gene sequences from 317 relevant cyanobacterial strains, which aided to our initial identification of kin strains of *Microcoleus asticus* sp. nov. IPMA8, for which we used the compressed set of 118 representative cyanobacterial strains. 16S rRNA gene sequences were separately aligned with MAFFT^91^ and trimmed using Gblocks v0.91b^92,93^. ML phylogeny values were calculated with IQTree^94^, using the best-fit model of substitution proposed by this algorithm, for 1.000 bootstraps under GTR + I + G model. Subsequently, to refine the phylogenetic relations of our strain and validate this gene tree, we calculated a phylogenetic tree of 12 Oscillatoriales strains using a set of 12 fully sequenced genomes, which was built from the concatenation of 64 gene sequences common to all 12 genomes (see supplementary Table S3). Gene sequences were separately aligned with MAFFT and trimmed using Gblocks v0.91b. To infer ML phylogenies, we used IQTree to compute and support the trees by calculating 1.000 bootstraps using GAMMA distribution and LG + I + F model, the best-fit model of substitution proposed by this algorithm. Bayesian phylogenies were inferred with MrBayes v3.2^95^ for 100.000 generations using the same model and a discarded burn-in rate of 25% of the initial generations. To further investigate the relationship of *Microcoleus asticus* sp. nov. IPMA8 among other Cyanobacteria from the Oscillatoriales order, we used the 16S rRNA gene sequence for a set 23 of publicly available cyanobacterial nucleic sequences. Trees were computed using RAxML under GTR + I + G model^96^, which we confirmed using PartitionFinder v.2.1.1^97^ for 500 bootstraps.

Genome similarity analysis were done by average nucleotide identity (ANI) using the online tool https://www.ezbiocloud.net/tools/ani ^38^ and DNA-DNA hybridization (DDH) using the online tool http://ggdc.dsmz.de/faq.php#qggdc24 ^39^.

### Analysis of the geosmin synthase gene in Bacteria

To interrogate the phylogenetic relationships of the geosmin synthase genes in Bacteria kingdom we inferred a gene tree with geosmin synthase genes available on Genbank (accessed on Oct. 2018). Candidate geosmin synthase genes were identified by BLAST comparison with a reference geosmin synthase gene from *Cylindrospermum stagnale* PCC 7417, with a cut-off value of 50% identity on similarity. We collected complete and partial sequences of geosmin synthase sequences from available databases (in the case of several *Nostoc* the gene was only partly sequenced) from 66 representative bacterial strains from four distinct phyla. Several putative gene sequences from the three bacterial phyla were identified, which including 36 cyanobacterial gene sequences, three Gammaproteobacteria, 14 Deltaproteobacteria and 13 Actinobacteria strains covering all identified genus and strains of known geosmin producers and non-producers. Gene sequences were aligned with MAFFT and trimmed with Gblocks resulting in an alignment of 273 amino acid positions, which included the N-terminal part of gene. This alignment was used to calculate the phylogenetic tree for geosmin synthase gene and a sesquiterpene cyclase (CAM04173.1) was used as outgroup^98^. To infer ML phylogenies, we used IQTree to calculate and support the trees for 1.000 bootstraps under LG + I + G4 model, the model proposed by this algorithm. To corroborate this tree, Bayesian phylogenies were calculated with MrBayes v3.2 for 100.000 generations using the same model and a discarded burn-in rate of 25% of the initial generations.

### Visualization of geosmin synthase gene sequence variation

To evaluate sequence similarities between the geosmin synthase genes, in our set of 66 Bacteria strains, we compared the calculated geosmin gene tree described in the previous section, with a heatmap representing pairwise sequence similarities values between all the gene sequences. This percent identity matrix was calculated, with the amino acid trimmed alignment, using CLUSTAL OMEGA online tool^99,100^ and the graphical representation was done with the online tool Evolview^101,102^.

### Detection of recombination

To detect adaptive evolution in the bacterial geosmin gene, the rates of synonymous and non-synonymous substitutions were estimated using algorithms in the Datamonkey.org web-server^103^. The detection possible recombination was performed with Genetic Algorithm for Recombination Detection (GARD)^104^, a method that identifies recombination, and can be used as a pre-analysis to the inference selection tests performed afterwards.

We used the protein alignment prepared for the geosmin gene distribution analysis in the 3 bacterial phyla which we back translated to DNA, as suggested by the software’s documentation, using EMBOSS Backtranseq V6.6.0 online tool^105^.

### Natural selection scanning

To detect the possibility of adaptive (positive) or purifying (negative) selection selection in the geosmin gene in Bacteria, we used Datamonkey web-server^103^ algorithms SLAC (Single-Likelihood Ancestor Counting) and FUBAR (Fast Unconstrained Bayesian AppRoximation). Finally, MEME (Mixed Effects Model of Evolution) performed a test for the identification of individual sites also subject to positive or purifying selection. The global ratio of non-synonymous nucleotide substitutions (dN) to synonymous nucleotide substitutions (dS) was employed as an indicator of positive or negative selection pressure. Diversifying or positive selection dominates when the value of dN/dS is >1, dN/dS >1 implies purifying or negative selection while dN/dS = 1 is suggestive of neutral selection pressure.

### Chemical analysis of geosmin production

The Cyanobacteria culture was tested for geosmin production using SPME GC-MS at iBET - Instituto de Biologia Experimental e Tecnológica by the Food and Health Division Laboratory (Oeiras, Portugal). Solid phase microextraction (SPME) was used for the extraction of volatile compounds from Cyanobacteria cultures. Briefly, 2.5 mL of sample (mix of fresh algae culture, 8 mL of ddH2O and 3 g of NaCl) were measured to a 10 mL headspace vial (La-Pha-Pack®) and were capped with a white PTFE silicone septum (Specanalitica). The SPME operating conditions were: extraction temperature 40 °C for 40 min, rotating speed 250 rpm, agitator during 10 s, desorption time 5 min at 260 °C. Analysis were carried out in a GCMS-QP2010 Plus (Shimadzu®) equipped with an AOC-5000 autosampler (Shimadzu®). A divinylbenzene/Carboxen/polydimethylsiloxane (DVB/Car/PDMS) fiber (SUPELCO Analytical, Bellefonte, PA, USA) was used for headspace SPME sampling. For the analysis a capillary column ZB-5MSi (Zebron, phenomenex®) capillary column 30 m, 0.25 mm (IS), 0.25 µm (film thickness) was used. The working conditions were: injector temperature: 260 °C, injection mode: splitless, detector temperature: 250 °C. High-purity helium (≥99.999%) was used as the carrier gas, column oven temperature was kept at 50 °C for 3 min, increased to 180 °C at a rate of 8 °C min^−1^ and maintained for 8 min, then was increased to 230 °C at 25 °C min^−1^ and maintained for 1 min.; carrier gas (He) 2.00 mL.min^−1^, interface and ion source temperature in MS were at 250 °C. Mass spectra were acquired in Electron Ionization (EI) mode at 70 eV. in a m/z range between 29–299 with a scan speed of 555 scans s^−1^. Geosmin in samples was detected in Single Ion Monitoring (SIM) mode using characteristic ions m/z 111, 112, 125 and 126. The geosmin solution (100 µg μL^−1^ in methanol; Sigma - Aldrich®) was used as standards for GC-MS analysis. Dilution series from 100 µg ml^−1^ to 5 ng ml^−1^ were prepared for the geosmin standard to test the response and sensitivity of the GC-MS method. Geosmin was identified using the mass spectra libraries, NIST 21, 27, 107, 147 and Wiley 229.

## Supplementary information

Supplementary Information.

## Data Availability

The nucleotide sequence data are available at DDBJ/EMBL/GenBank under the accession number: PRJNA531034. The Holotype was deposit in the Herbarium of University of Coimbra (COI), Coimbra, Portugal (http://www.uc.pt/en/herbario_digital/) in a metabolically inactive state in the form of preserved material with the strain identifiers (COI00097100; COI00097101; COI00097102; COI00097103) and the information avalilable online at: https://coicatalogue.uc.pt/index.php. Cultures of *Microcoleus asticus* were deposited in in the Collection of Microalgae of University of Coimbra (ACOI) at the Department of Life Sciences, University of Coimbra, Coimbra, Portugal and at the Culture Collection of Cyanobacteria of the Portuguese Institute for Sea and Atmosphere, I. P. (IPMA, IP), Av. Alfredo Magalhães Ramalho, 6, 1495–165 Algés under the strain identifiers ACOI 3416 and IPMA8 respectively. Phycobank registration http://phycobank.org/102090.

## References

[1] Mazard S, Penesyan A, Ostrowski M, Paulsen I, Egan S (2016). Tiny Microbes with a Big Impact: The Role of Cyanobacteria and Their Metabolites in Shaping Our Future. Mar. Drugs.

[2] Schirrmeister BE, Gugger M, Donoghue PCJ (2015). Cyanobacteria and the Great Oxidation Event: Evidence from genes and fossils. Palaeontology.

[3] Swain SS, Paidesetty SK, Padhy RN (2017). Antibacterial, antifungal and antimycobacterial compounds from cyanobacteria. Biomed. Pharmacother..

[4] Shishido TK (2015). Antifungal compounds from cyanobacteria. Mar. Drugs.

[5] Nowruzi B, Haghighat S, Fahimi H, Mohammadi E (2018). *Nostoc* cyanobacteria species: a new and rich source of novel bioactive compounds with pharmaceutical potential. J. Pharm. Heal. Serv. Res..

[6] Blom JF (2006). Potent algicides based on the cyanobacterial alkaloid nostocarboline. Org. Lett..

[7] Blagojević D (2018). Antioxidant activity and phenolic profile in filamentous cyanobacteria: the impact of nitrogen. J. Appl. Phycol..

[8] He X (2016). Toxic cyanobacteria and drinking water: Impacts, detection, and treatment. Harmful Algae.

[9] Bláha L, Babica P, Maršálek B (2009). Toxins produced in cyanobacterial water blooms - toxicity and risks. Interdiscip. Toxicol..

[10] Lee J, Rai PK, Jeon YJ, Kim KH, Kwon EE (2017). The role of algae and cyanobacteria in the production and release of odorants in water. Environ. Pollut..

[11] Watson SB, Monis P, Baker P, Giglio S (2016). Biochemistry and genetics of taste- and odor-producing cyanobacteria. Harmful Algae.

[12] Pattanaik B, Lindberg P (2015). Terpenoids and Their Biosynthesis in Cyanobacteria. Life.

[13] Godo T (2017). Geosmin-producing Species of *Coelosphaerium* (Synechococcales, Cyanobacteria) in Lake Shinji, Japan. Sci. Rep..

[14] Liato V, Aïder M (2017). Chemosphere Geosmin as a source of the earthy-musty smell in fruits, vegetables and water: Origins, impact on foods and water, and review of the removing techniques. Chemosphere.

[15] Zaitlin B, Watson SB (2006). Actinomycetes in relation to taste and odour in drinking water: Myths, tenets and truths. Water Res..

[16] Schrader KK, Summerfelt ST (2009). Distribution of Off-Flavor Compounds and Isolation of Geosmin-Producing Bacteria in a Series of Water Recirculating Systems for Rainbow Trout. Culture. N. Am. J. Aquac..

[17] Paterson, R. R. M., Venâncio, A. & Lima, N. Why do food and drink smell like earth? *Commun. Curr. Res. Educ. Top. Trends Appl. Microbiol*. 120–128 (2007).

[18] Lu G, Edwards CG, Fellman JK, Scott Mattinson D, Navazio J (2003). Biosynthetic origin of geosmin in red beets (*Beta vulgaris* L.). J. Agric. Food Chem..

[19] Schlumpberger BO, Jux A, Kunert M, Boland W, Wittmann D (2004). Musty-Earthy Scent in Cactus Flowers: Characteristics of Floral Scent Production in Dehydrogeosmin-Producing Cacti. Int. J. Plant Sci..

[20] Ogura T, Sunairi M, Nakajima M (2000). 2-Methylisoborneol and Geosmin, the Main Sources of Soil Odor, Inhibit the Germination of Brassicaceae Seeds. Soil Sci. Plant Nutr..

[21] Kanchiswamy, C. N., Malnoy, M. & Maffei, M. E. Chemical diversity of microbial volatiles and their potential for plant growth and productivity. *Front. Plant Sci*. **6**, (2015). 10.3389/fpls.2015.00151 PMC4358370 25821453

[22] War, A. R. *et al*. Psb-7-1306. **7**, 1306–1320 (2012).

[23] Wang Z, Xiao P, Song G, Li Y, Li R (2015). Isolation and characterization of a new reported cyanobacterium *Leptolyngbya bijugata* coproducing odorous geosmin and 2-methylisoborneol. Environ. Sci. Pollut. Res..

[24] Suurnäkki S (2015). Identification of geosmin and 2-methylisoborneol in cyanobacteria and molecular detection methods for the producers of these compounds. Water Res..

[25] Juttner F, Watson SB (2007). Biochemical and Ecological Control of Geosmin and 2-Methylisoborneol in Source Waters. Appl. Environ. Microbiol..

[26] Liaimer A, Jensen JB, Dittmann E (2016). A genetic and chemical perspective on symbiotic recruitment of cyanobacteria of the genus *Nostoc* into the host plant *Blasia pusilla* L. Front. Microbiol..

[27] Teodoro-Morrison T, Diamandis EP, Rifai N (2014). Animal Olfactory Detection of Disease: Promises and Pitfalls. Am. Assoc. Clin. Chem..

[28] Tosi L, Sola C (1993). Role of Geosmin, a Typical Inland Water Odour, in Guiding Glass Eel *Anguilla anguilla* (L.) Migration. Ethology.

[29] Stensmyr MC (2012). A conserved dedicated olfactory circuit for detecting harmful microbes in drosophila. Cell.

[30] Jiang J, He X, Cane DE (2007). Biosynthesis of the earthy odorant geosmin by a bifunctional *Streptomyces coelicolor* enzyme. Nat. Chem. Biol..

[31] Cane DE, Ikeda H (2012). Exploration and Mining of the Bacterial Terpenome. Acc. Chem. Res..

[32] Christianson DW (2006). Structural Biology and Chemistry of the Terpenoid Cyclases. Chem. Rev..

[33] Giglio S, Jiang J, Saint CP, Cane DE, Monis PT (2008). Isolation and characterization of the gene associated with geosmin production in cyanobacteria. Environ. Sci. Technol..

[34] Wang Z, Shao J, Xu Y, Yan B, Li R (2015). Genetic basis for geosmin production by the water bloom-forming cyanobacterium, *Anabaena ucrainica*. Water.

[35] Hug LA (2016). A new view of the tree of life. Nat. Microbiol..

[36] Bolotin E, Hershberg R (2015). Gene loss dominates as a source of genetic variation within clonal pathogenic bacterial species. Genome Biol. Evol..

[37] Harris GG (2015). Structural Studies of Geosmin Synthase, a Bifunctional Sesquiterpene Synthase with αα Domain Architecture That Catalyzes a Unique Cyclization–Fragmentation Reaction Sequence. Biochemistry.

[38] Yoon SH, Ha SM, Lim JM, Kwon SJ, Chun J (2017). A large-scale evaluation of algorithms to calculate average nucleotide identity. Antonie van Leeuwenhoek.

[39] Meier-Kolthoff JP (2013). Genome sequence-based species delimitation with confidence intervals and improved distance functions. BMC Bioinformatics.

[40] Turland, N. J. *et al*. (eds.): International Code of Nomenclature for algae, fungi, and plants (Shenzhen Code) adopted by the Nineteenth International Botanical Congress Shenzhen, China, July 2017. Regnum Vegetabile 159. Glashütten: Koeltz Botanical Books, 10.12705/Code (2018).

[41] Strunecký O, Komárek J, Johansen J, Lukešová A, Elster J (2013). Molecular and morphological criteria for revision of the genus *Microcoleus* (Oscillatoriales, Cyanobacteria). J. Phycol..

[42] Palinska KA, Marquardt J (2008). Genotypic and phenotypic analysis of strains assigned to the widespread cyanobacterial morphospecies *Phormidium autumnale* (Oscillatoriales). Arch. Microbiol..

[43] Starkenburg SR (2011). Genome of the cyanobacterium *Microcoleus vaginatus* FGP-2, a photosynthetic ecosystem engineer of arid land soil biocrusts worldwide. J. Bacteriol..

[44] Komárek J, Kastovsky J, Mares J, Johansen JR (2014). Taxonomic classification of cyanoprokaryotes (cyanobacterial genera) 2014, using a polyphasic approach. Preslia.

[45] Strunecky O, Komarek J, Šmarda J (2014). Kamptonema (Microcoleaceae, Cyanobacteria), a new genus derived from the polyphyletic *Phormidium* on the basis of combined molecular and cytomorphological markers. Preslia.

[46] Tindall BJ, Rosselló-Móra R, Busse HJ, Ludwig W, Kämpfer P (2010). Notes on the characterization of prokaryote strains for taxonomic purposes. Int. J. Syst. Evol. Microbiol..

[47] Kim M, Oh HS, Park SC, Chun J (2014). Towards a taxonomic coherence between average nucleotide identity and 16S rRNA gene sequence similarity for species demarcation of prokaryotes. Int. J. Syst. Evol. Microbiol..

[48] Nevo R (2007). Thylakoid membrane perforations and connectivity enable intracellular traffic in cyanobacteria. EMBO J..

[49] Nevo Reinat, Chuartzman Silvia G., Tsabari Onie, Reich Ziv, Charuvi Dana, Shimoni Eyal (2009). Architecture of Thylakoid Membrane Networks. Lipids in Photosynthesis.

[50] Mareš J, Strunecký O, Bučinská L, Wiedermannová J (2019). Evolutionary Patterns of Thylakoid Architecture in Cyanobacteria. Front. Microbiol..

[51] Boyer, S. L., Johansen, J. R., Flechtner, V. R., Howard, G. L. & Bliss, F. Phylogeny and genetic variance in terrestrial *Microcoleus* (cyanophyceae) species based on sequence analysis of the 16s rrna gene and associated 16s–23s its region. **1235**, 1222–1235 (2002).

[52] Williams L, Loewen-Schneider K, Maier S, Büdel B (2016). Cyanobacterial diversity of western European biological soil crusts along a latitudinal gradient. FEMS Microbiol. Ecol..

[53] Dvořák P, Hašler P, Poulíčková A (2012). Phylogeography of the *Microcoleus vaginatus* (Cyanobacteria) from Three Continents – A Spatial and Temporal Characterization. PLoS One.

[54] Garcia-Pichel F, Lopez-Cortes A, Nubel U (2001). Phylogenetic and Morphological Diversity of Cyanobacteria in Soil Desert Crusts from the Colorado Plateau. Appl. Environ. Microbiol..

[55] Sergeeva E, Liaimer A, Bergman B (2002). Evidence for production of the phytohormone indole-3-acetic acid by cyanobacteria. Planta.

[56] Ghalab NM, Tantawy EA, Khalil HMA, Shaban KA (2016). Plant Growth Promoters Substances that Excreting from Bacteria and Cyanobacteria as Essential Factors for Alleviation Soil Salt Stress on Rice Plant. J. Microbiol. Res..

[57] Lan S, Wu L, Zhang D, Hu C (2014). Desiccation provides photosynthetic protection for crust cyanobacteria *Microcoleus vaginatus* from high temperature. Physiol. Plant..

[58] Bu C, Wu S, Yang Y, Zheng M (2014). Identification of Factors Influencing the Restoration of Cyanobacteria-Dominated Biological Soil Crusts. PLoS One.

[59] Rossi F, De Philippis R (2015). Role of Cyanobacterial Exopolysaccharides in Phototrophic Biofilms and in Complex Microbial Mats. Life.

[60] Rossi F, Adessi A, De Philippis R (2016). Biological soil crusts: from ecology to biotechnology. Perspect. Phycol..

[61] Ludwig F (2007). Identification and expression analyses of putative sesquiterpene synthase genes in *Phormidium* sp. and prevalence of *geo*A-like genes in a drinking water reservoir. Appl. Environ. Microbiol..

[62] Zhou A (2012). Functional characterization of Crp/Fnr-type global transcriptional regulators in *Desulfovibrio vulgaris* hildenborough. Appl. Environ. Microbiol..

[63] Asquith EA, Evans CA, Geary PM, Dunstan RH, Cole B (2013). The role of Actinobacteria in taste and odour episodes involving geosmin and 2-methylisoborneol in aquatic environments. J. Water Supply Res. Technol..

[64] Wang C (2013). Antifungal activity of volatile organic compounds from *Streptomyces alboflavus* TD-1. FEMS Microbiol. Lett..

[65] Agger SA, Lopez-Gallego F, Hoye TR, Schmidt-Dannert C (2008). Identification of Sesquiterpene Synthases from *Nostoc punctiforme* PCC 73102 and *Nostoc* sp. Strain PCC 7120. J. Bacteriol..

[66] Newton RJ, Jones SE, Eiler A, McMahon KD, Bertilsson S (2011). A Guide to the Natural History of Freshwater Lake Bacteria. Microbiol. Mol. Biol. Rev. Mar..

[67] Li ZF (2011). Genome sequence of the halotolerant marine bacterium *Myxococcus fulvus* HW-1. J. Bacteriol..

[68] Dávila-Céspedes A, Hufendiek P, Crüsemann M, Schäberle TF, König GM (2016). Marine-derived myxobacteria of the suborder Nannocystineae: An underexplored source of structurally intriguing and biologically active metabolites. *Beilstein*. J. Org. Chem..

[69] Sánchez-Baracaldo P (2015). Origin of marine planktonic cyanobacteria. Sci Rep.

[70] Wang Z (2019). The diversity, origin, and evolutionary analysis of geosmin synthase gene in cyanobacteria. Science of The Total Environment.

[71] Rantala-Ylinen A (2004). Phylogenetic evidence for the early evolution of microcystin synthesis. Proceedings of the National Academy of Sciences.

[72] Hoefel D (2006). Cooperative biodegradation of geosmin by a consortium comprising three gram-negative bacteria isolated from the biofilm of a sand filter column. Letters in Applied Microbiology.

[73] Hoefel D, Ho L, Monis PT, Newcombe G, Saint CP (2009). Biodegradation of geosmin by a novel Gram-negative bacterium; isolation, phylogenetic characterisation and degradation rate determination. Water Research.

[74] Zhou B (2011). Biodegradation of geosmin in drinking water by novel bacteria isolated from biologically active carbon. Journal of Environmental Sciences.

[75] Guttman L, van Rijn J (2012). Isolation of bacteria capable of growth with 2-methylisoborneol and geosmin as the sole carbon and energy sources. Applied and Environmental Microbiology.

[76] Marmulla R, Harder J (2014). Microbial monoterpene transformations-a review. Frontiers in Microbiology.

[77] Siegesmund MA, Johansen JR, Karsten U, Friedl T (2008). *Coleofasciculus* gen. nov. (Cyanobacteria): Morphological and molecular criteria for revision of the genus *Microcoleus* Gomont. J. Phycol..

[78] Churro C (2009). Effects of bacillamide and newly synthesized derivatives on the growth of cyanobacteria and microalgae cultures. J. Appl. Phycol..

[79] Semedo-Aguiar AP, Pereira-Leal JB, Leite RB (2018). Microbial Diversity and Toxin Risk in Tropical Freshwater Reservoirs of Cape Verde. Toxins (Basel)..

[80] Bankevich A (2012). SPAdes: A New Genome Assembly Algorithm and Its Applications to Single-Cell Sequencing. J. Comput. Biol..

[81] Gurevich A, Saveliev V, Vyahhi N, Tesler G (2013). QUAST: quality assessment tool for genome assemblies. Bioinformatics.

[82] Seemann T (2014). Prokka: Rapid prokaryotic genome annotation. Bioinformatics.

[83] Aziz RK (2008). The RAST Server: rapid annotations using subsystems technology. BMC Genomics.

[84] Huerta-Cepas J (2016). eggNOG 4.5: a hierarchical orthology framework with improved functional annotations for eukaryotic, prokaryotic and viral sequences. Nucleic Acids Res..

[85] Bland C (2007). CRISPR Recognition Tool (CRT): a tool for automatic detection of clustered regularly interspaced palindromic repeats. BMC Bioinformatics.

[86] Kall L, Krogh A, Sonnhammer ELL (2007). Advantages of combined transmembrane topology and signal peptide prediction–the Phobius web server. Nucleic Acids Res..

[87] Arndt D (2016). PHASTER: a better, faster version of the PHAST phage search tool. Nucleic Acids Res..

[88] Simão FA, Waterhouse RM, Ioannidis P, Kriventseva EV, Zdobnov EM (2015). BUSCO: Assessing genome assembly and annotation completeness with single-copy orthologs. Bioinformatics.

[89] Blin K, Medema MH, Kottmann R, Lee SY, Weber T (2017). The antiSMASH database, a comprehensive database of microbial secondary metabolite biosynthetic gene clusters. Nucleic Acids Res..

[90] Ramos V, Morais J, Vasconcelos VM (2017). A curated database of cyanobacterial strains relevant for modern taxonomy and phylogenetic studies. Sci. Data.

[91] Katoh K, Standley DM (2013). MAFFT multiple sequence alignment software version 7: Improvements in performance and usability. Mol. Biol. Evol..

[92] Castresana J (2000). Selection of Conserved Blocks from Multiple Alignments for Their Use in Phylogenetic Analysis. Mol. Biol. Evol..

[93] Talavera G, Castresana J (2007). Improvement of phylogenies after removing divergent and ambiguously aligned blocks from protein sequence alignments. Syst. Biol..

[94] Nguyen LT, Schmidt HA, Von Haeseler A, Minh BQ (2015). IQ-TREE: A fast and effective stochastic algorithm for estimating maximum-likelihood phylogenies. Mol. Biol. Evol..

[95] Ronquist F (2012). Mrbayes 3.2: Efficient bayesian phylogenetic inference and model choice across a large model space. Syst. Biol..

[96] Stamatakis A (2014). RAxML version 8: a tool for phylogenetic analysis and post-analysis of large phylogenies. Bioinformatics.

[97] Lanfear R, Frandsen PB, Wright AM, Senfeld T, Calcott B (2017). Partitionfinder 2: New methods for selecting partitioned models of evolution for molecular and morphological phylogenetic analyses. Mol. Biol. Evol..

[98] Komatsu M, Tsuda M, Omura S, Oikawa H, Ikeda H (2008). Identification and functional analysis of genes controlling biosynthesis of 2-methylisoborneol. Proc. Natl. Acad. Sci. USA.

[99] Sievers F (2014). Fast, scalable generation of high-quality protein multiple sequence alignments using Clustal Omega. Mol. Syst. Biol..

[100] Li W (2015). The EMBL-EBI bioinformatics web and programmatic tools framework. Nucleic Acids Res..

[101] He Z (2016). Evolview v2: an online visualization and management tool for customized and annotated phylogenetic trees. Nucleic Acids Res..

[102] Zhang H, Gao S, Lercher MJ, Hu S, Chen WH (2012). EvolView, an online tool for visualizing, annotating and managing phylogenetic trees. Nucleic Acids Res..

[103] Weaver Steven, Shank Stephen D, Spielman Stephanie J, Li Michael, Muse Spencer V, Kosakovsky Pond Sergei L (2018). Datamonkey 2.0: A Modern Web Application for Characterizing Selective and Other Evolutionary Processes. Molecular Biology and Evolution.

[104] Pond SLK, Posada D, Gravenor MB, Woelk CH, Frost SDW (2006). GARD: a genetic algorithm for recombination detection. Bioinformatics..

[105] Madeira F (2019). The EMBL-EBI search and sequence analysis tools APIs in 2019. Nucleic Acids Research.

[106] Hoiczyk E, Hansel A (2000). Cyanobacterial cell walls: News from an unusual prokaryotic envelope. J. Bacteriol..

[107] Hoiczyk E, Baumeister W (1997). Oscillin, an extracellular, Ca2+ -binding glycoprotein essential for the gliding motility of cyanobacteria. Mol. Microbiol..

