# Supplementary material for: A novel cyanobacterial geosmin producer, revising GeoA distribution and dispersion patterns in Bacteria
Source: Sci Rep. 2020 May 26;10:8679. doi: 10.1038/s41598-020-64774-y (PMC7251104; doi:10.1038/s41598-020-64774-y)
Supplement: Supplementary file 1 — Supplementary Information. [file 41598_2020_64774_MOESM1_ESM.docx]

**Supplementary Information**

Title: A novel cyanobacterial geosmin producer, revising GeoA distribution and dispersion patterns in Bacteria.

Authors: Catarina Churro; Ana P. Semedo-Aguiar; Alexandra D. Silva; Jose B. Pereira-Leal and Ricardo B. Leite

**
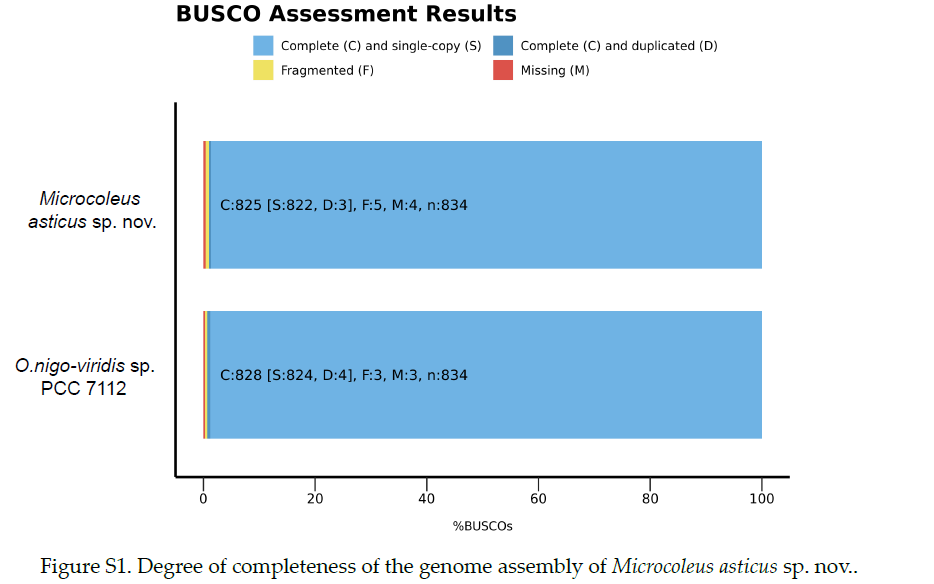
**


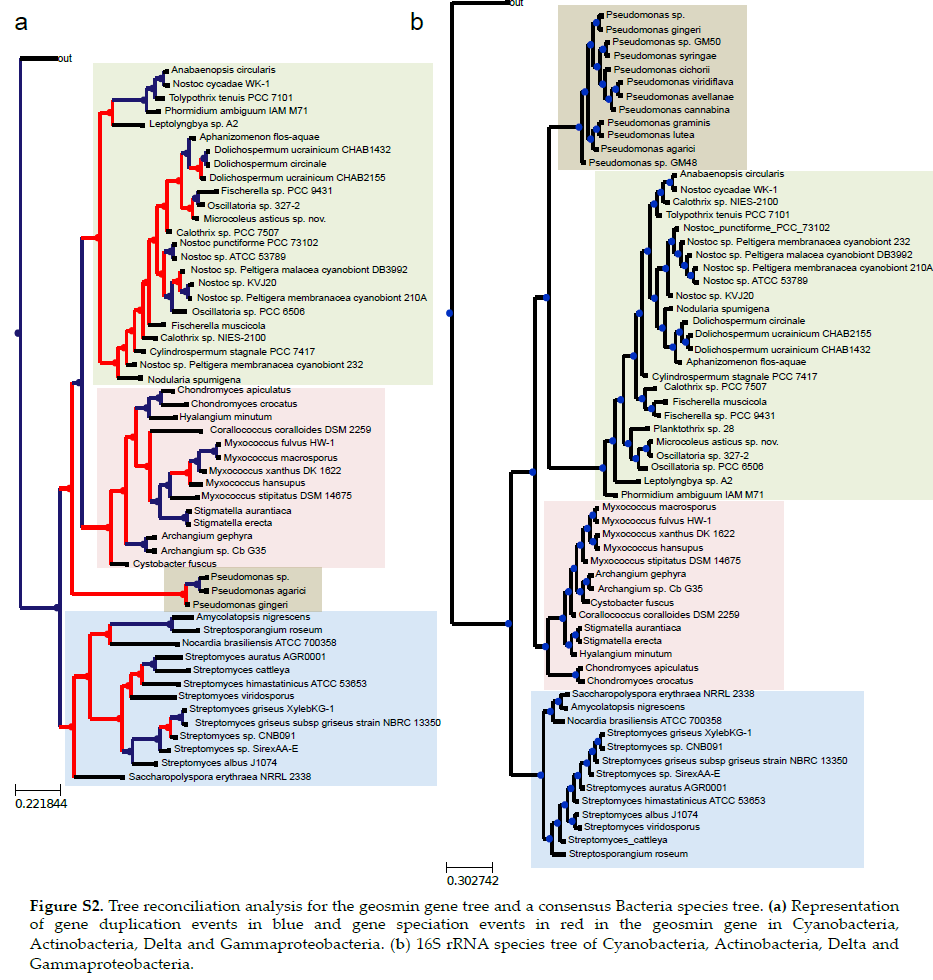


Table S1 – RAST functional annotation of the predicted transcriptome of *Microcoleus asticus* sp. nov.
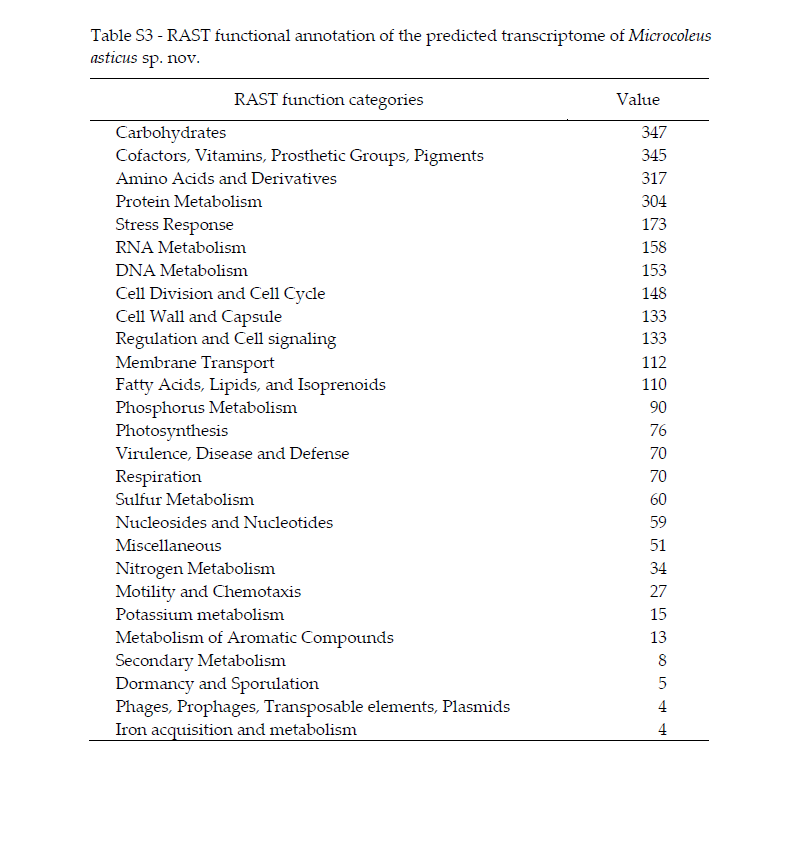


Table S2. Description of the Bacterial strains used in this study.

| **Species** | **Strain** | **Geosmin production** | **Habitat/origin** | **Taxonomic Group (order)** | ***geo*A**  **Accession Number** |
| --- | --- | --- | --- | --- | --- |
| *Nostoc punctiforme* | PCC 73102 / ATCC® 29133™ | Yes^1,2^ | Terrestrial, Symbiont, *Macrozamia* sp. root (cycad), Australia, 1973^3^ | Nostocales | WP_012409287.1 |
| *Nostoc* sp. | MB5357/ ATCC® 53789™ | Yes^4^ | Terrestrial, Symbiont, Lichen, Scotland: Arron Island^5^ | Nostocales | AIZ06050.1 |
| *Nostoc* sp. | DB3992 | No info. | Terrestrial, Symbiont, Lichen, *Peltigera malacea*, Canada: Little Fort, British Columbia, 2008 (SAMN07192327) | Nostocales | WP_099100688.1 |
| *Nostoc* sp. | 210A | No info. | Terrestrial, Symbiont, Lichen, *Peltigera membranacea*, Iceland: Thveras 21, Reykjavik, 2012 (SAMN07191894) | Nostocales | WP_094347146.1 |
| *Nostoc* sp. | 232 | No info. | Terrestrial, Symbiont, Lichen, *Peltigera membranacea*, Iceland: Eldgja, brink, 2012 (SAMN07191897) | Nostocales | WP_094343825.1 |
| *Nostoc* sp. | UK1 | Yes^4^ | Terrestrial, Symbiont, Lichen, *Peltigera membranacea*, Finland: Itä-Pakila^4^ | Nostocales | AIZ06054.1 |
| *Nostoc* sp. | UK4 | Yes^4^ | Terrestrial, Symbiont, Lichen, *Peltigera membranacea*, Finland: Itä-Pakila^4^ | Nostocales | AIZ06052.1 |
| *Nostoc* sp. | UK3 | Yes ^4^ | Terrestrial, Symbiont, Lichen, *Peltigera membranacea*, Finland: Itä-Pakila^4^ | Nostocales | AIZ06053.1 |
| *Nostoc* sp. | 268 | Yes^4^ | Unknown, Russia^4^ | Nostocales | AIZ06051.1 |
| *Nostoc* sp. | KVJ20 | No info. | Terrestrial, Symbiont, Plant, *Blasia pusilla*, Norway: Troms, Kvaloya island, 2006^6^ (SAMN04453661) | Nostocales | WP_069072178.1 |
| *Calothrix* sp. | PCC 7507/ ATCC® 29112™ | Yes ^4, 1, 7^ | Terrestrial, Free living, Moss carpet, *Sphagnum* bog, Switzerland: Kastanienbaum^3^ | Nostocales | WP_015129218.1 |
| *Aphanizomenon* sp. | PMC9501 | Yes^4, 7^ | Freshwater, Free living, France: Lake Chambon, 1995^8^ | Nostocales | AIZ06049.1 |
| *Aphanizomenon*  *flos-aquae* | NIES-81 | No info. | Freshwater, Free living, Japan: Lake Kasumigaura, 1978 (SAMN02485748 ) | Nostocales | WP_027402321.1 |
| *Aphanizomenon*  *gracile* | WH - 1 | Yes | Freshwater, Free living, China: Wuhan (AJZ76763.1) | Nostocales | AJZ76763.1 |
| *Anabaena* sp. | CRKS33 | No info. | Freshwater, USA: Cheney Reservoir, WA, 2013 (SAMN04028824) | Nostocales | OBQ38409.1 |
| *Dolichospermum ucrainicum* | CHAB2155 | Yes^9^ | Freshwater, Free living, China: Erhai lakes^9^ | Nostocales | AEA03341.1 |
| *Dolichospermum ucrainicum* | CHAB1432 | Yes^9^ | Freshwater, Free living, China: Lake Dianchi^9^ | Nostocales | AEA03338.1 |
| *Dolichospermum*  *circinale* | AWQC131C | Yes^10, 11^ | Freshwater, Free living,  Australia: New South Wales, Lake Cargelligo, Lachlan River, 1991 (SAMN02471998) | Nostocales | WP_028089363.1 |
| *Phormidium* sp. | P2r_1 | Yes^10^ | Freshwater, Free living, Germany: Saidenbach drinking water reservoir | Oscillatoriales | ABU93238.1 |
| *Phormidium* sp. | P2r_2 | Yes^10^ | Freshwater, Free living, Germany: Saidenbach drinking water reservoir | Oscillatoriales | ABU93239.1 |
| *Oscillatoria* sp. | 327/2 | Yes^4^ | Freshwater, Free living, Aquarium, Finland^4^ | Oscillatoriales | AIZ06055.1 |
| *Fischerella* sp. | PCC 9431 | No info. | Unknown (SAMN02441755) | Nostocales | WP_026720761.1 |
| *Planktothrix* sp. | 328 | Yes^4^ | Freshwater, Free living, Aquarium, Finland^4^ | Oscillatoriales | AIZ06056.1 |
| *Oscillatoria* sp. | PCC 9240/ Oscillatoria 193 | Yes^4^ | Freshwater, Free living, Finland: Lake Hormajärvi, 1986^12^ | Oscillatoriales | AIZ06057.1 |
| *Oscillatoria* sp. | PCC 6506/ATCC® 29081™/UTEX 1547 | Yes^4^ | Unknown^3^ | Oscillatoriales | AFZ61536.1 |
| *Fischerella muscicola* | PCC 73103/ SAG 1427-1/ATCC 29114 | Yes^13^ | Terrestrial, Free living, India 1951: rice field^3^ | Nostocales | WP_016859507.1 |
| *Calothrix* sp. | NIES 2100/IAM M-56/Ishikawa 78. F243D | No info. | Unknown (SAMD00079806) | Nostocales | WP_096600485.1 |
| *Cylindrospermum*  *stagnale* | PCC 7417 | Yes^9^ | Terrestrial, Free living, Sweeden: Stockholm, 1972 (SAMN02261342) | Nostocales | WP_015209723.1 |
| *Nodularia spumigena* | CCY9414 | No info. | Brackish water, Free living, Denmark: Bornholm, Baltic Sea (SAMN01915661) | Nostocales | WP_063873980.1 |
| *Tolypothrix tenuis* | PCC7101/ATCC® 27914™ | No info. | Terrestrial, Free living, soil sample, Borneo, 1950^3^ | Nostocales | BAY99334.1 |
| *Nostoc* sp. | NIES2111/ IAM M-272/KK-01 | No info. | Terrestrial, Free living,soil sample, Japan: Kakuma Campus, Kanazawa University (SAMD00079809) | Nostocales | WP_096683179.1 |
| *Anabaenopsis*  *circularis* | NIES21/IAM M-4 | No info. | Terrestrial, Free living, soil sample, Origin unknown^14^ (SAMD00079795) | Nostocales | BAY16576.1 |
| *Nostoc cycadae* | WK-1 | No info. | Terrestrial, Symbiont, *Cycas revoluta*, Japan: Wakayama, Wakayama University, 1977 (SAMD00053836) | Nostocales | GBE94253.1 |
| *Phormidium*  *ambiguum* | IAM M-71 / NIES-2119 | No info. | Freshwater, Free living, Brazil^15^ | Oscillatoriales | WP_084555082.1 |
| *Nostoc linckia* | NIES25/IAM M-16 (=M-251) | No info. | Terrestrial, Free living, Japan: Kagoshima^16^ (SAMD00079798) | Nostocales | WP_099072309.1 |
| *Leptolyngbya*  *bijugata* | A2 | Yes^9^ | Freshwater, Free living, China: river in Xiaogan, Hubei Province, 2012^9^ | Synechococcales | AJP00077.1 |
| *Amycolatopsis nigrescens* | CSC17Ta-90 | No info. | Terrestrial (SAMN02261308) | Actinomycetales | WP_020667245.1 |
| *Archangium gephyra* | ATCC25201/DSM 2261 | No info. | Terrestrial (SAMN04488083) | Myxococcales | WP_047855443.1 |
| *Archangium* sp. | Cb_G35 | No info. | Tree bark (SAMN06020410) | Myxococcales | WP_073566941.1 |
| *Chondromyces apiculatus* | DSM_436 | No info. | Decayed wood (PRJNA192263) | Myxococcales | WP_044243564.1 |
| *Chondromyces crocatus* | Cm_c5 | No info. | Terrestrial SAMN03651481 | Myxococcales | WP_050434199.1 |
| *Corallococcus coralloides* | DSM 2259 | No info. | Terrestrial (SAMN02603593) | Myxococcales | AFE08479.1 |
| *Cystobacter fuscus* | DSM 2262 | No info. | Terrestrial (SAMN02470006) | Myxococcales | WP_043432513.1 |
| *Hyalangium minutum* | DSM 14724 | No info. | Terrestrial, soil with decaying plant material (SAMN02719563) | Myxococcales | WP_044199666.1 |
| *Myxococcus fulvus* | HW-1 | No info. | Marine (SAMN02603152) | Myxococcales | AEI64954.1 |
| *Myxococcus hansupus* | Cm_a2/ex DSM436 | No info. | Decayed wood (SAMN02469988) | Myxococcales | AKQ63738.1 |
| *Myxococcus macrosporus* | DSM 14697 | No info. | Terrestrial (SAMN06167052) | Myxococcales | WP_095960569.1 |
| *Myxococcus stipitatus* | DSM 14675 | No info. | Terrestrial (SAMN02603594) | Myxococcales | AGC48036.1 |
| *Myxococcus xanthus* | DK 1622 | Yes | Terrestrial (SAMN02604018) | Myxococcales | ABF90553.1 |
| *Nocardia brasiliensis* | ATCC 700358 | No info. | Human mycetoma (SAMN02603853) | Actinomycetales | AFU00809.1 |
| *Pseudomonas agarici* | NCPPB 2289 | No info. | On *Agaricus bisporus*, mushroom (SAMN02471740) | Pseudomonadales | WP_060782878.1 |
| *Pseudomonas gingeri* | NCPPB 3146 | No info. | On *Agaricus bisporus*, ginger blotch disease in mushroos (SAMN02471742) | Pseudomonadales | WP_017126613.1 |
| *Pseudomon*as sp. | QS1027 | No info. | Soil (SAMN08100099) | Pseudomonadales | WP_100938764.1 |
| *Saccharopolyspora*  *erythraea* | NRRL 2338/  ATCC 11635 | Yes | Terrestrial (SAMN02470651) | Actinomycetales | WP_009945636.1 |
| *Stigmatella aurantiaca* | DW4_3-1/  DSM 17044 | Yes | Tree bark (SAMN05444354) | Myxococcales | WP_075006513.1 |
| *Stigmatella erecta* | DSM 16858 | No info | Tree bark (SAMN05443639) | Myxococcales | WP_093525208.1 |
| *Streptomyces albus* | J1074 | Yes | Straw | Actinomycetales | EFE84188.1 |
| *Streptomyces auratus* | AGR0001 | No info | Terrestrial (SAMN02472162) | Actinomycetales | EJJ06545.1 |
| *Streptomyces cattleya* | NRRL 8057/DSM 46488 | No info | Terrestrial (SAMN02603879) | Actinomycetales | WP_014143690.1 |
| *Streptomyces griseus* | NBRC 13350 | Yes | Terrestrial (SAMN03863253) | Actinomycetales | WP_012382258.1 |
| *Streptomyces griseus* | XylebKG-1 | No info | Beetle associated, (*Xyleborinus saxeseni*) (SAMN00713595) | Actinomycetales | ZP_08240572.1 |
| *Streptomyces*  *himastatinicus* | ATCC53653 | No info | Terrestrial (SAMN02595299) | Actinomycetales | EFL24857.1 |
| *Streptomyces* sp. | CNB091 | No info | Unknown (SAMN02194545) | Actinomycetales | WP_018956438.1 |
| *Streptomyces* sp. | SirexAA-E | No info | Adult Sirex woodwasp associated (SAMN03863220) | Actinomycetales | AEN08192.1 |
| *Streptomyces viridosporus* | ATCC 14672 | No info | Terrestrial (SAMN02595233) | Actinomycetales | WP_004981892.1 |
| *Streptosporangium roseum* | NRRL B-2638 | Yes | Terrestrial, paddy field soil (SAMN02645241) | Actinomycetales | WP_031158422.1 |

References used for Table S1:

1. Agger, S. A., Lopez-Gallego, F., Hoye, T. R. & Schmidt-Dannert, C. Identification of Sesquiterpene Synthases from *Nostoc punctiforme* PCC 73102 and *Nostoc* sp. Strain PCC 7120. *J. Bacteriol.* **190,** 6084–6096 (2008).

2. Giglio, S., Jiang, J., Saint, C. P., Cane, D. E. & Monis, P. T. Isolation and characterization of the gene associated with geosmin production in cyanobacteria. *Environ. Sci. Technol.* **42,** 8027–32 (2008).

3. Stanier, R. Y., Deruelles, J., Rippka, R., Herdman, M. & Waterbury, J. B. Generic Assignments, Strain Histories and Properties of Pure Cultures of Cyanobacteria. *Microbiology* **111,** 1–61 (1979).

4. Suurnäkki, S. *et al.* Identification of geosmin and 2-methylisoborneol in cyanobacteria and molecular detection methods for the producers of these compounds. *Water Res.* **68,** 56–66 (2015).

5. American Type Culture Collection (ATCC). Available at: https://www.lgcstandards-atcc.org/Products/All/53789.aspx#generalinformation.

6. Sundman, P. *et al.* Molecular characterization of planktic cyanobacteria of *Anabaena*, *Aphanizomenon*, *Microcystis* and *Planktothrix* genera. *Int. J. Syst. Evol. Microbiol.* **51,** 513–526 (2015).

7. Höckelmann, C., Becher, P. G. & Reuß, S. H. Von. Sesquiterpenes of the Geosmin-Producing Cyanobacterium *Calothrix* PCC 7507 and their Toxicity to Invertebrates. *Z. Naturforsch.* **64,** 49–55 (2009).

8. Gugger, M. Phylogenetic comparison of the cyanobacterial genera *Anabaena* and *Aphanizomenon*. *Int. J. Syst. Evol. Microbiol.* **52,** 1867–1880 (2002).

9. Wang, Z., Shao, J., Xu, Y., Yan, B. & Li, R. Genetic basis for geosmin production by the water bloom-forming cyanobacterium, *Anabaena ucrainica*. *Water* **7,** 175–187 (2015).

10. Tsao, H. W. *et al.* Monitoring of geosmin producing *Anabaena circinalis* using quantitative PCR*. Water Res.* ***49,*** *416–425 (2014).*

11. Watson, S. B., Monis, P., Baker, P. & Giglio, S. Biochemistry and genetics of taste- and odor-producing cyanobacteria. *Harmful Algae* **54,** 112–127 (2016).

12. Araoz, R. Neurotoxins in axenic oscillatorian cyanobacteria: coexistence of anatoxin-a and homoanatoxin-a determined by ligand-binding assay and GC/MS. *Microbiology* **151,** 1263–1273 (2005).

13. Wu, J. T. & Jüttner, F. Effect of Environmental Factors on Geosmin Production by *Fischerella muscicola*. *Water Sci. Technol.* **20,** 143–148 (1988).

14. Microbial Culture Collection at the National Institute for Environmental Studies (NIES). Available at: http://mcc.nies.go.jp/strainList.do?strainId=18&strainNumberEn=NIES-21.

15. Zhu, T. *et al.* crossm Draft Genome Sequences of Nine Cyanobacterial Strains from Diverse. 1–2 (2017).

16. Microbial Culture Collection at the National Institute for Environmental Studies (NIES). Available at: http://mcc.nies.go.jp/strainList.do?strainId=172&strainNumberEn=NIES-25.

Table S3:
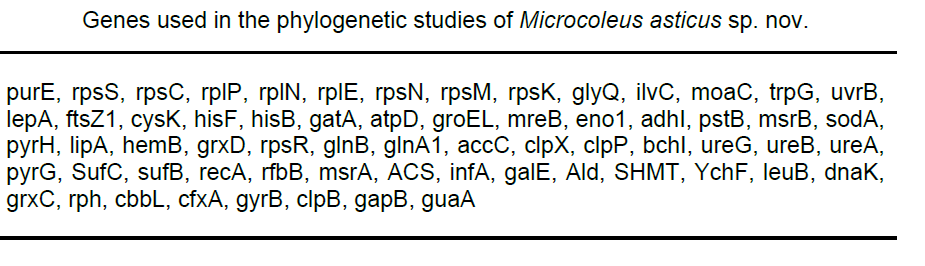


Table S4. Percent Similarity matrix results from analysis of the alignment of 64 genes present in *Oscillatoria nigro-viridis* PCC 7112, *Microcoleus asticus* IPMA8 and *Microcoleus vaginatus* FGP-2.

| % | *Oscillatoria nigro-viridis* PCC 7112 | *Microcoleus asticus* IPMA8 | *Microcoleus vaginatus* FGP-2 |
| --- | --- | --- | --- |
| *Oscillatoria nigro-viridis* PCC 7112 | 100.00 |  |  |
| *Microcoleus asticus* IPMA8 | 93.17 | 100.00 |  |
| *Microcoleus vaginatus* FGP-2 | 94.27 | 97.01 | 100.00 |
